# Generalizable prediction of childhood ADHD symptoms from neurocognitive testing and youth characteristics

**DOI:** 10.1038/s41398-023-02502-6

**Published:** 2023-06-24

**Authors:** Alexander Weigard, Katherine L. McCurry, Zvi Shapiro, Meghan E. Martz, Mike Angstadt, Mary M. Heitzeg, Ivo D. Dinov, Chandra Sripada

**Affiliations:** 1 Department of Psychiatry, University of Michigan, Ann Arbor, USA; 2 Department of Psychology, Emory University, Atlanta, USA; 3 Departments of Computational Medicine and Bioinformatics, and Health Behavior and Biological Sciences, University of Michigan, Ann Arbor, USA

**Keywords:** Human behaviour, Learning and memory, ADHD, Predictive markers

## Abstract

Childhood attention-deficit/hyperactivity disorder (ADHD) symptoms are believed to result from disrupted neurocognitive development. However, evidence for the clinical and predictive value of neurocognitive assessments in this context has been mixed, and there have been no large-scale efforts to quantify their potential for use in generalizable models that predict individuals’ ADHD symptoms in new data. Using data drawn from the Adolescent Brain Cognitive Development Study (ABCD), a consortium that recruited a diverse sample of over 10,000 youth (ages 9–10 at baseline) across 21 U.S. sites, we develop and test cross-validated machine learning models for predicting youths’ ADHD symptoms using neurocognitive abilities, demographics, and child and family characteristics. Models used baseline demographic and biometric measures, geocoded neighborhood data, youth reports of child and family characteristics, and neurocognitive tests to predict parent- and teacher-reported ADHD symptoms at the 1-year and 2-year follow-up time points. Predictive models explained 15–20% of the variance in 1-year ADHD symptoms for ABCD Study sites that were left out of the model-fitting process and 12–13% of the variance in 2-year ADHD symptoms. Models displayed high generalizability across study sites and trivial loss of predictive power when transferred from training data to left-out data. Features from multiple domains contributed meaningfully to prediction, including neurocognition, sex, self-reported impulsivity, parental monitoring, and screen time. This work quantifies the information value of neurocognitive abilities and other child characteristics for predicting ADHD symptoms and provides a foundational method for predicting individual youths’ symptoms in new data across contexts.

## Introduction

Attention-deficit/hyperactivity disorder (ADHD) is a common psychiatric diagnosis of childhood characterized by difficulty maintaining focus, disorganization, impulsivity, and excessive movement in inappropriate contexts [1]. Although the formal diagnosis of ADHD has guided most extant research and clinical practice, there is growing acknowledgment that symptoms of ADHD likely reflect a dimensional trait that varies across the entire population [2–4]. ADHD symptoms in youth have been linked to many negative outcomes, including poorer academic achievement [5], substance use and externalizing behavior [6], and persistent financial difficulties [7].

As influential theoretical models posit that aberrations in neurocognitive development are a central cause of childhood ADHD symptoms [8, 9], measures of neurocognitive performance have been widely used in both etiological research [10–12] and clinical practice [13]. However, the evidence base for such measures is mixed. Although medium- to large-sized effects of ADHD diagnosis on neurocognitive performance are commonly observed in group comparisons [14, 15], individuals with ADHD cannot be effectively distinguished using neurocognitive data alone [16–19]. Furthermore, evidence that such measures display disadvantages relative to subjective rating scales for predicting ADHD symptoms and associated impairment [20–23] has driven ongoing debate about whether cognitive measures contain useful information for characterizing or predicting the ADHD phenotype [13, 19, 24, 25].

Against this backdrop, current evidence for the relevance of neurocognitive performance to childhood ADHD symptoms exhibits several serious gaps. Studies examining predictors from multiple measurement domains “head-to-head” are essential for determining whether neurocognitive data reduce uncertainty about the clinical phenotype to a non-trivial degree over and above contributions from other domains and therefore add value for etiological research and clinical management. However, comparative studies are relatively rare and typically use limited sets of measures [19–22], making it difficult to comprehensively quantify the information value of different measurement domains. Also notably lacking in this area are studies that use machine learning and cross-validation methods to systematically evaluate the performance of predictive models using independent data (i.e., data that were not used to train the model). Such tests are critical for establishing the robustness and generalizability of predictive models when applied to the broader population [26], which is essential for harnessing neurocognitive and other predictors to inform research and clinical practices across diverse settings.

To address these gaps, we turned to the Adolescent Brain Cognitive Development Study (ABCD) [27, 28], a large-scale longitudinal consortium study of over 10,000 U.S. children who were recruited at ages 9–10. ABCD presents an unprecedented opportunity for developing predictive models of ADHD symptoms due to its size and inclusion of diverse communities that make up the U.S. population. Furthermore, ABCD’s consortium structure, in which data are collected at 21 different sites across the U.S., provides a natural “leave-one-site-out” cross-validation approach for evaluating whether models generalize to unseen study sites that vary in their geographic location, participant demographics, and investigative teams [29].

In the current study, we leverage ABCD to develop and test generalizable machine learning models for predicting ADHD symptoms in independent data using neurocognitive testing and features from other relevant measurement domains (demographics, geocoding, and child reports of personality, family structure, and social context). We index ADHD symptoms at the baseline, 1-year, and 2-year follow-up time points using a multi-rater approach, considered the gold standard for minimizing rater biases and measuring impairments across contexts [30, 31]. As individuals’ ADHD symptoms tend to display high temporal stability across this developmental period [32–35], we primarily focus on predicting individuals’ trait levels of ADHD symptoms rather than longitudinal changes. Leave-one-site-out cross-validation is used to assess the accuracy of multivariate predictive models in unseen data and quantify the predictive contributions of neurocognitive testing versus other measurement domains.

## Subjects and methods

### Sample and ADHD symptom data

Data were from the curated data release version 4.0 of the ABCD Study (https://nda.nih.gov; 10.15154/1,523,041), a large-scale consortium that recruited 11,878 children, ages 9–10, across 21 study sites and is following them throughout their adolescence. Participants were recruited with a sampling strategy designed to closely reflect the demographic diversity of the U.S., as described elsewhere [28]. The study protocol was approved by local institutional review boards at each site. Parents and caregivers provided written informed consent child participants provided verbal assent.

Given the importance of considering multiple raters when assessing ADHD [30, 31], we used both parent-report data from the Child Behavior Checklist (CBCL) [36, 37] and teacher-report data from the Brief Problem Monitor (BPM) [38] to construct the ADHD symptoms variable. However, as teacher-report data from roughly half or more of the ABCD sample were missing at each wave (due to factors including non-responses or parents declining permission to contact teachers), these missing data had to be accounted for. Recent work in the ABCD sample on the evidence-based definition of ADHD [39] used multiple imputation to replace missing teacher-report data based on evidence that imputation is unlikely to inflate effects of interest and, at worst, will underestimate effects. As parent-report data, which we used to impute the missing teacher-report data in ABCD, typically have only a modest relation with teacher reports [31], we took several steps to gauge the impact of the imputation procedure on our measurement and predictions of ADHD symptoms. As described in Supplementary Material, we determined that the factor loadings Supplementary Tables 1 and 2), fit indices, and factor scores (Supplementary Fig. 1) of the cross-rater ADHD symptoms measurement model (detailed below) were generally robust to imputation. We also conducted all primary analyses focused on predicting ADHD symptoms at the 1-year timepoint twice: first within all individuals who met our broader inclusion criteria with missing symptom data imputed as needed using the *mice* [40] R package (“full” sample) and second only within individuals who had complete data from both parents and teachers at the 1-year timepoint (“complete data” sample). As detailed below, the general pattern of findings was similar between these analyses.

We focused on predicting symptoms at the 1-year timepoint because the number of individuals without missing ADHD symptom data (*n* = 5915 prior to other exclusions) was substantially greater than at either the baseline (*n* = 3960) or 2-year (*n* = 4164) time points. Although the aim of the study was to predict individuals’ ADHD symptom level as a relatively stable trait rather than to predict changes in symptoms across time points, we also conducted analyses aimed at gauging how changes across time points relate to the features and accuracy of the predictive models. Specifically, we predicted ADHD symptom data at the 2-year timepoint to assess how much prediction suffers when the symptom measurements are further temporally separated from the baseline measures used in the model. We then computed residual change scores (Δ) by regressing individuals’ ADHD symptoms scores at the 1-year and 2-year time points, separately, on their baseline symptoms. Residuals from these regressions reflect changes in individuals’ symptoms that occurred from the baseline to these time points. We then attempted to predict both sets of residual change scores.

Participants were excluded from analyses if they (1) had siblings across different ABCD sites at baseline (which compromises test data independence in cross-validation), (2) were unaffiliated with one of the main 21 sites at baseline, (3) were missing all parent- and teacher-report items used to measure ADHD symptoms, or (4) were one of 300 unrelated individuals randomly selected for an independent subsample used to generate priors for computational cognitive modeling of baseline cognitive tasks. These exclusion procedures left 11,530 individuals with available ADHD symptom data at baseline, 10,933 individuals at the 1-year timepoint, and 10,104 individuals at the 2-year timepoint, with reductions in the later time points reflecting attrition in ABCD. The “complete data” subsample of participants with complete parent- and teacher-report ADHD symptom data at the 1-year timepoint consisted of 5900 individuals. For analyses predicting residual difference scores, 10,931 individuals had both baseline and 1-year data, while 10,103 individuals had both baseline and 2-year data.

### Cross-rater measure of ADHD symptoms

We fit a bifactor structural equation model to parent-report items from the Attention Problems Syndrome scale and ADHD DSM-oriented scale of the CBCL and teacher-report items from the Attention/Hyperactivity Problems scale of the BPM using the R package *lavaan* [41]. This method allows common variance in ADHD symptoms indicated across raters—the outcome of interest—to be measured via a general factor while rater-specific variance is accounted for by orthogonal parent- and teacher-report subfactors. To prevent data bleed in cross-validation, we re-estimated the model in each training fold (which involved repeating the multiple imputation step for analyses involving missing symptom report data) and used the estimated model to generate symptom scores in both the training fold and the associated test data. Consistent with the well-known stability in the rank-ordering of children’s ADHD symptoms across development [32–35], we found that the ADHD symptoms factor showed correlations of 0.66 to 0.77 across study time points. Hence, the ADHD symptom measure reflects a relatively stable trait during this developmental period. The measurement model’s development, loadings, fit, and temporal stability are described in Supplementary Material.

### Predictor variables

To prevent predictive models from becoming optimistically biased by shared-method variance [42] or rater biases, we excluded parent and teacher rating scales from consideration as predictive features. Instead, we focused on objective (e.g., demographics, geocoding), child-reported, and neurocognitive test features. The 54 features from the baseline timepoint that were used in predictive models are summarized here and further detailed in Supplementary Material and Supplementary Tables 3–5.

Basic demographic features included age, sex, race, ethnicity, parental marital status, parental income, and parental education. Geocoded variables inferred from the Census tract of the child’s primary residence [43] included neighborhood poverty, as indexed by an Area Deprivation Index aggregate measure developed in prior work [44]; total crimes; lead exposure risk; and measures of educational quality and opportunity (e.g., high-school graduation rate). We also included the basic biometric variables of body mass index (BMI) and waist circumference.

Child self-report features included measures of personal, family, and system-level characteristics. Children’s self-reported impulsivity was measured by the BIS/BAS [45] and UPPS [46]. Children’s perceptions of parental monitoring, family conflict, their school system, and neighborhood crime were derived from ABCD culture and environment assessments [47]. Children’s estimates of their time spent engaged in screen media on typical weekdays and weekend days were obtained from the ABCD Screen Time Questionnaire [48, 49].

Neurocognitive features included age-corrected scores from all seven subtests of the NIH Toolbox, which were designed to span the cognitive domains of episodic memory, working memory, attention, processing speed, and verbal ability [50]. They also included total memory recall from the Rey Auditory Verbal Learning Test (RAVLT) [51], age-corrected scores from the Matrix Reasoning Task [52], accuracy rate from the Little Man Task of visuo-spatial processing [53], and the single-item Cash Choice Task of delay of gratification [54]. We included parameters of the diffusion decision model (DDM) [55] estimated using trial-level data from tasks administered during neuroimaging: the stop-signal task (SST) and n-back. The DDM is a computational model that allows the estimation of parameters indexing specific mechanisms of cognitive performance, including cognitive efficiency (“drift rate”), caution in responding (“boundary separation”), and perceptual/motor processes (“nondecision time”), some of which have been found to relate to ADHD [12]. The DDM described the data from all tasks well (Supplementary Figs. 2–4)

### Machine learning and cross-validation procedures for predicting ADHD symptoms

We used two modeling strategies that take complementary approaches toward extracting predictive information from intercorrelated feature sets. The first, principal components regression (PCR) [56], involves using principal components analysis (PCA) [57] to identify a smaller number of dimensions that can explain individual variation in the set of predictive features and then entering individuals’ expression scores for each component into a linear regression. The second, least absolute shrinkage and selection operator regression (LASSO) [58, 59], includes a regularization parameter, λ, that penalizes regression coefficients for less important features, causing them to either be reduced or excluded from the model altogether.

Each approach has distinct strengths. PCR provides a *comprehensive* predictive model representing latent dimensions that explain intercorrelation across all available features. PCR can therefore be used to estimate the degree of predictive information individual features contain regardless of their intercorrelations. LASSO instead generates a *sparse* predictive model by excluding redundant features. In this way, the inclusion of a feature in LASSO is a clear indication that it contributes meaningfully to prediction, but the exclusion of a feature by LASSO does not necessarily mean it lacks predictive information, as it could contain information that is valuable but redundant with interrelated features. We, therefore, interpreted results from PCR to gauge the predictive value of individual features regardless of their possible redundancy and used LASSO to develop more parsimonious models.

We used leave-one-site-out cross-validation [29] to train all models and test their generalizability. For each left-out ABCD site, predictive models were trained in data from all other sites and used to generate predicted values for the ADHD symptoms score in both the training data and the held-out test site. Missing data were addressed with multiple imputation models estimated in training data with the *mice* package. The correlation coefficient (*r*) between predicted and actual symptom scores in the test data was used to gauge model accuracy. To further prevent our analytic decisions from biasing conclusions, we set aside data from three ABCD sites in a ‘lockbox’ prior to variable selection and only evaluated model performance in lockbox sites once all other analyses were finalized. Procedures for model estimation and cross-validation are detailed in Supplementary Material and the analysis code is available on the Open Science Framework: https://osf.io/zf82n/.

### Interpretation of feature and domain contributions to prediction

Feature contributions to prediction were quantified with feature weights, averaged across the models trained in all 18 iterations of the leave-one-site-out cross-validation procedure. Weights were computed for each feature in the comprehensive (PCR) models by multiplying the matrix of feature loadings on each component by the matrix of components’ linear regression coefficients from the predictive model.

For the sparse (LASSO) modeling method, we aimed to assess feature weights from the most parsimonious model possible. As LASSO can be unstable across samples, we only included features in this final LASSO model that were both (1) included by LASSO across all 18 training and (2) included across both halves of a split-half analysis in which LASSO was fit to data from nine sites, drawn at random, and separately fit to data from the other nine sites. We evaluated whether this parsimonious model, made up of only the most robust features, performed similarly to models in which all features were entered. We then averaged this model’s regression coefficients across training folds to generate feature weights.

To quantify the relative predictive power of variables from neurocognitive, child self-report, and demographic (including geocoded and biometric) measurement domains for predicting individuals’ trait ADHD symptoms, we alternately evaluated model performance when the variables in each domain were exclusively used for prediction, as well as when they were deliberately left out.

## Results

### Predictive models of trait ADHD symptoms show high generalizability to unseen data across ABCD sites

**Fig. 1:**
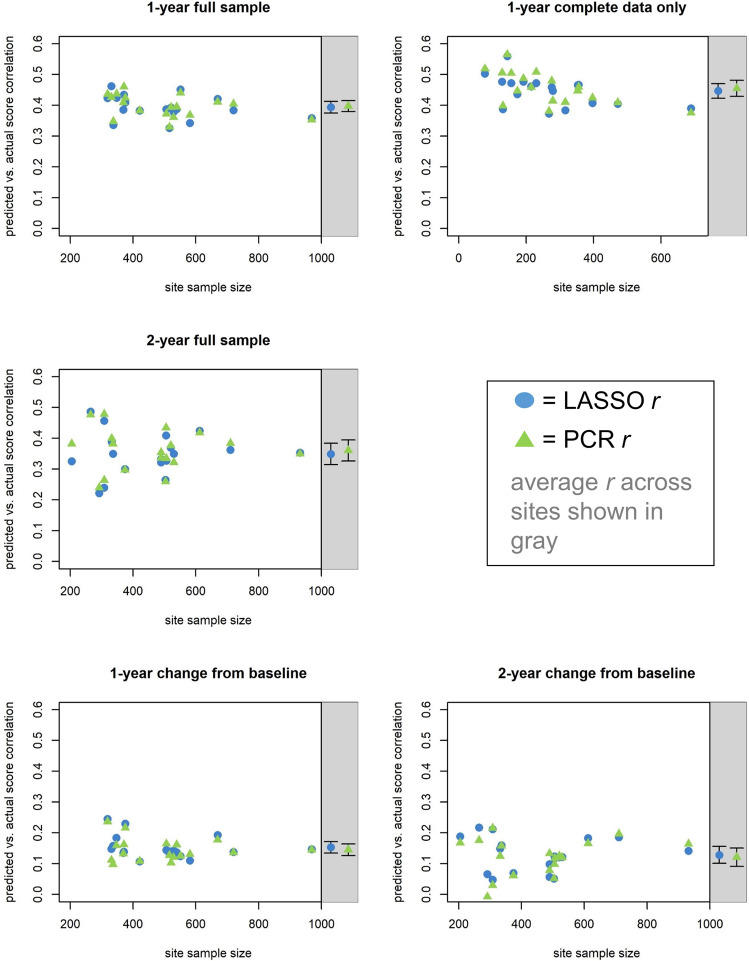
Accuracy and generalizability of predictive models. For each application of predictive modeling, plots display site sample sizes and correlation coefficients (*r*) for the relation between predicted and actual ADHD symptom measures in all 18 sites when data from each site were left out of the respective training set. The top row displays *r* values for models predicting ADHD symptoms at the 1-year time point in the full sample with missing symptom data imputed (left) and in the subsample of individuals with complete data (right). The middle row displays *r* values for models predicting 2-year symptoms, and the bottom row displays *r* values for models predicting changes in symptoms from baseline. Green triangles represent models that use principal component regression (PCR), while blue circles represent those that use LASSO. The shaded region at right shows the average *r* values across sites and their 95% confidence intervals (CIs).

**Fig. 2:**
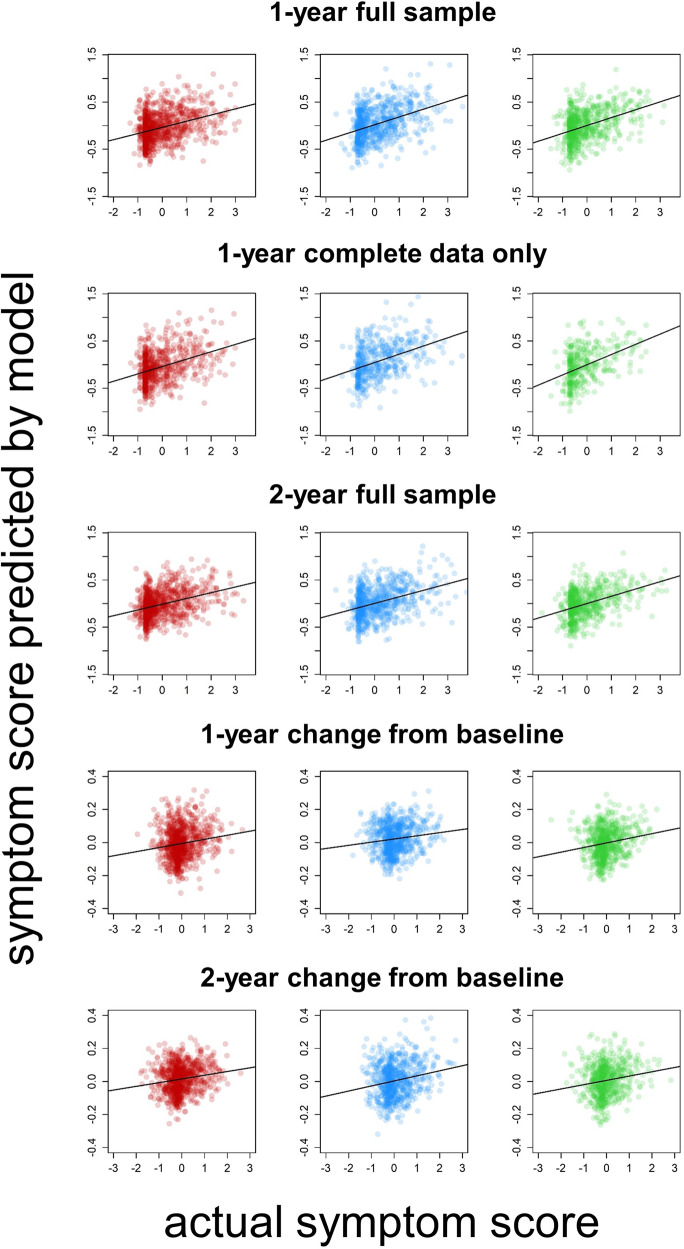
Comparisons of predicted and actual symptom scores. Scatterplots depict the relation between predicted and actual symptom scores in the three study sites with the largest sample sizes for all reported principal components regression (PCR) analyses involving the full matrix of predictive features.

Figure 1 displays correlation (*r*) values for relations between actual and predicted ADHD symptoms in the 18 sites included in the primary analyses when each site was left out of its respective model-training data set. Figure 2 shows scatterplots of these relations for the three sites with the largest sample size. Prediction of ADHD symptoms at the 1-year time point was generally consistent across ABCD sites and was nearly identical, on average, across the comprehensive (PCR) and sparse (LASSO) predictive modeling methods. For the “full data” sample with missing ADHD symptom data imputed (*n* = 8972), average *r* values across left-out sites (PCR *r* = 0.40, CI = 0.38–0.42; LASSO *r* = 0.39, CI = 0.38–0.41) indicated that the models explained roughly 15–16% of the variance in symptoms. For the “complete data” sample that included only individuals with complete ADHD symptom data (*n* = 4855), the models explained roughly 20% of the variance in left-out sites (PCR *r* = 0.46, CI = 0.43–0.48; LASSO *r* = 0.45, CI = 0.42–0.47). Remarkably, in both cases, the level of model performance in left-out sites was nearly identical to the average model performance within the training folds (full data PCR = 0.41, LASSO = 0.41; complete data PCR = 0.46, LASSO = 0.45), indicating the models were highly generalizable to unseen data.

Results were similar in sensitivity analyses in which children who were prescribed stimulant medication at the time of their baseline visit were excluded from the full sample with imputed symptom data (8.0% excluded; PCR *r* = 0.38, CI = 0.36–0.40; LASSO *r* = 0.37, CI = 0.36–0.39) and from the sample of individuals with complete symptom data (8.2% excluded; PCR *r* = 0.44, CI = 0.41–0.46; LASSO *r* = 0.43, CI = 0.40–0.46). Hence, the overall efficacy of the models was broadly robust to possible medication effects on predictive features. Furthermore, when we evaluated model performance in the three lockbox sites, we found the models were similarly accurate, both for the full sample with imputed symptom data (PCR *r* = {0.41,0.39,0.38}; LASSO *r* = {0.42,0.37,0.36}) and for the sample of only individuals with complete symptom data (PCR *r* = {0.46,0.44,0.45}; LASSO *r* = {0.47,0.40,0.41}).

We also evaluated whether the modeling methods were similarly effective at predicting ADHD symptoms at the 2-year time point (with all missing ADHD symptom data imputed, given the lower proportion of individuals with complete data at this time point). The resulting models were able to explain 12–13% of the variance in ADHD symptoms for sites left out of the model-fitting process (PCR *r* = 0.36, CI = 0.33–0.39; LASSO *r* = 0.35, CI = 0.31–0.38) and continued to show high generalizability across the primary sites (Fig. 1) and across the three lockbox sites (PCR *r* = {0.39,0.37,0.41}; LASSO *r* = {0.39,0.36,0.39). Given that the temporal separation between the baseline data and 2-year ADHD symptom measures was twice that of the primary analyses, this represents a modest reduction in predictive performance when compared to the 1-year sample with imputed data (15–16%) and likely reflects the fact that the ADHD symptoms measure itself shows a high degree of temporal stability across study waves.

### Features with the most predictive information are a mixture of neurocognitive, demographic, and child self-report variables

**Fig. 3:**
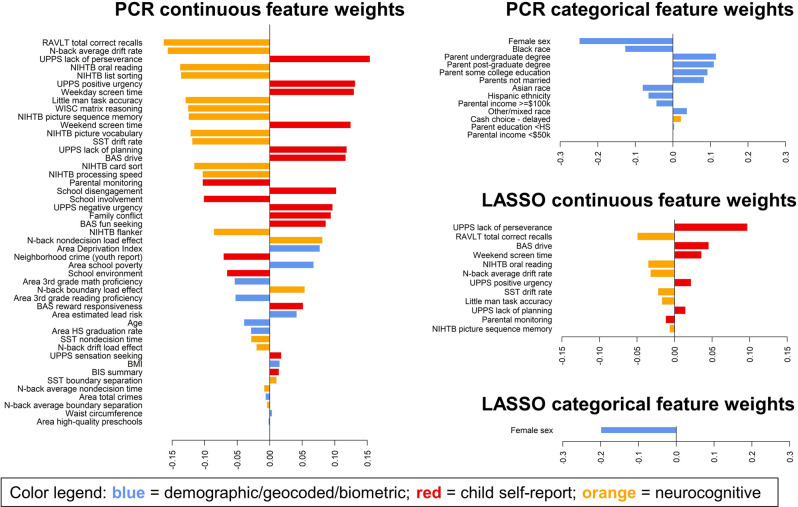
Feature weights for models predicting ADHD symptoms at the 1-year timepoint in the full sample with missing symptom data imputed. The bar plots display feature weights, averaged across training folds and ranked by absolute value, for the comprehensive PCR models and the sparse LASSO models. The sparse LASSO model only included 13 of the most relevant features (i.e., those consistently selected in all training folds as well as both halves of the split-half analysis). Weights are color-coded by variable domain: blue = demographic/geocoded/biometric, red = child self-report, orange = neurocognitive testing. Continuous features are shown separately from categorical features because the predictor variables were scaled differently; continuous features were *z*-scored (mean = 0, SD = 1), while categorical features were coded as dummy variables with 0 = reference group versus 1 = feature present. RAVLT Rey Auditory Verbal Learning Test, NIHTB NIH Toolbox, WISC Wechsler Intelligence Scale for Children, SST Stop Signal Task, UPPS Impulsive Behavior Scale, BIS Behavioral Inhibition System, BAS Behavioral Approach System, HS High School, BMI body mass index.

Feature weights from the comprehensive (PCR) models predicting 1-year ADHD symptoms in the full sample (Fig. 3) represent each feature’s degree of predictive information, regardless of whether that information is unique to this feature or part of a broader latent dimension of individual variability (i.e., is also reflected in other features). These weights indicated that most neurocognitive indices, including measures of reasoning, memory, verbal ability, processing speed, and the DDM’s cognitive efficiency (“drift rate”) parameter, showed strong negative predictive relations with ADHD symptoms. Neurocognitive features with relatively weak weights included other DDM parameters and measures of n-back cognitive load effects. Child self-report features indicated that greater impulsivity, greater screen time, lower parental monitoring, greater family conflict, and lower involvement/engagement in school were most predictive of symptoms. Of the basic demographics, male sex played an outsized role in prediction. Of the geocoded variables, measures of neighborhood poverty (Area Deprivation Index and school poverty) were the most predictive. In contrast, measures of lead exposure risk and neighborhood crime contributed relatively little. Biometric measures also contributed little.

These patterns of feature weights were highly consistent with those in analyses of the subsample of individuals with complete 1-year symptom data (Supplementary Fig. 5) and analyses predicting symptoms at the 2-year time point (Supplementary Fig. 6), suggesting that predictive features were robust to missing data imputation and outcome measurement timepoint.

### A parsimonious model including only neurocognition, sex, impulsivity, parental monitoring, and screen time achieves near-maximal predictive performance

When forming the sparse LASSO model for the primary analysis predicting 1-year ADHD symptoms in the sample with missing symptom data imputed, we found that only 13 features were consistently selected across all training folds and both halves of the split-half analysis. The final sparse LASSO model that included only these 13 features displayed a performance that was practically identical to the more complex PCR and LASSO models in which all features were entered (*r* = 0.39, CI = 0.37–0.41). It also performed only slightly worse than the more complex models when applied to the lockbox sites (*r*s = {0.41,0.37,0.35}). Hence, this sparse modeling strategy selected a parsimonious set of important features that achieve near-maximal predictive accuracy. These 13 key features (Fig. 3) included sex, six neurocognitive measures (tests of multiple cognitive domains and the DDM cognitive efficiency parameters), screen time, parental monitoring, and children’s self-reported impulsivity. Most of the same features, with the exception of parental monitoring, were also included in the sparse model for the sample with complete ADHD symptom data at the 1-year time point and the sparse model for predicting ADHD symptom data at the 2-year time point (Supplementary Figs. 5, 6), suggesting that they are robustly predictive of ADHD symptoms.

### Neurocognitive testing meaningfully boosts predictive power

When used on their own, data from the neurocognitive, child self-report, and demographic (including geocoded and biometric) domains explained roughly 7%, 10%, and 6% of the variance in 1-year symptoms, respectively, in unseen data when the full 1-year sample was considered (Table 1). When neurocognitive, child self-report, and demographic data were selectively left out of analyses, the resulting models explained roughly 12%, 11%, and 14% of the variance, respectively, indicating that information from all three measurement domains was necessary to achieve maximal performance (16%). In the subset of individuals with complete 1-year symptom ratings, neurocognitive, child self-report, and demographic data explained roughly 10%, 13%, and 7% of the variance (Table 2). When neurocognitive, child self-report, and demographic data were selectively left out of these analyses, the resulting models explained roughly 16%, 15%, and 18% of the variance, respectively, compared to 20% for the model with information from all three domains. Therefore, neurocognitive data appear to explain roughly 4% of the variance over and above other domains, and this added value is consistent across the analyses of the full sample and subset with complete symptom data.

**Table 1:** Average model performance across training folds and across held-out test sites, as indexed by the correlation coefficient (*r*) for the relation between predicted attention problems and actual attention problems at the 1-year time point in the full sample of individuals with missing symptom data imputed.

Predictors	PCR models			LASSO models		
	Training *r* mean	Test *r* mean	Test *r* CI	Training *r* mean	Test *r* mean	Test *r* CI
Full predictor set	0.41	0.40	0.38–0.42	0.41	0.39	0.38–0.41
Sparse/LASSO-selected predictors	-	-	-	0.40	0.39	0.37–0.41
Neurocognitive data only	0.28	0.26	0.24–0.29	0.27	0.26	0.24–0.28
Child self-report data only	0.33	0.32	0.29–0.34	0.32	0.31	0.29–0.33
Demographic data only	0.25	0.24	0.20–0.27	0.24	0.23	0.20–0.26
Neurocognitive data left out	0.37	0.35	0.32–0.38	0.36	0.35	0.32–0.38
Child self-report data left out	0.34	0.33	0.31–0.35	0.33	0.33	0.31–0.34
Demographic data left out	0.38	0.37	0.35–0.39	0.37	0.36	0.34–0.38

Confidence intervals (95% CIs) are reported for the mean *r* value across the 18 test sites. CIs are not computed for the mean *r* value across training folds because of the large degree of overlap between folds, which makes them non-independent.

*PCR* principal components regression, *LASSO* least absolute shrinkage and selection operator regression.

**Table 2:** Average model performance across training folds and across held-out test sites, as indexed by the correlation coefficient (*r*) for the relation between predicted attention problems and actual attention problems at the 1-year time point in the sample of individuals who had complete symptom data.

Predictors	PCR models			LASSO models		
	Training *r* mean	Test *r* mean	Test *r* CI	Training *r* mean	Test *r* mean	Test *r* CI
Full predictor set	0.46	0.46	0.43–0.48	0.45	0.45	0.42–0.47
Sparse/LASSO-selected predictors	-	-	-	0.45	0.45	0.42–0.47
Neurocognitive data only	0.32	0.31	0.27–0.34	0.31	0.29	0.25–0.33
Child self-report data only	0.36	0.36	0.33–0.38	0.35	0.35	0.32–0.38
Demographic data only	0.29	0.27	0.24–0.30	0.28	0.27	0.25–0.30
Neurocognitive data left out	0.41	0.39	0.37–0.42	0.40	0.40	0.38–0.42
Child self-report data left out	0.39	0.39	0.36–0.41	0.39	0.38	0.36–0.40
Demographic data left out	0.42	0.42	0.39–0.46	0.42	0.41	0.38–0.44

Confidence intervals (95% CIs) are reported for the mean *r* value across the 18 test sites. CIs are not computed for the mean *r* value across training folds because of the large degree of overlap between folds, which makes them non-independent.

*PCR* principal components regression, *LASSO* least absolute shrinkage and selection operator regression.

### Models predicting residual symptom change scores have lower accuracy but highlight similar predictive features

**Fig. 4:**
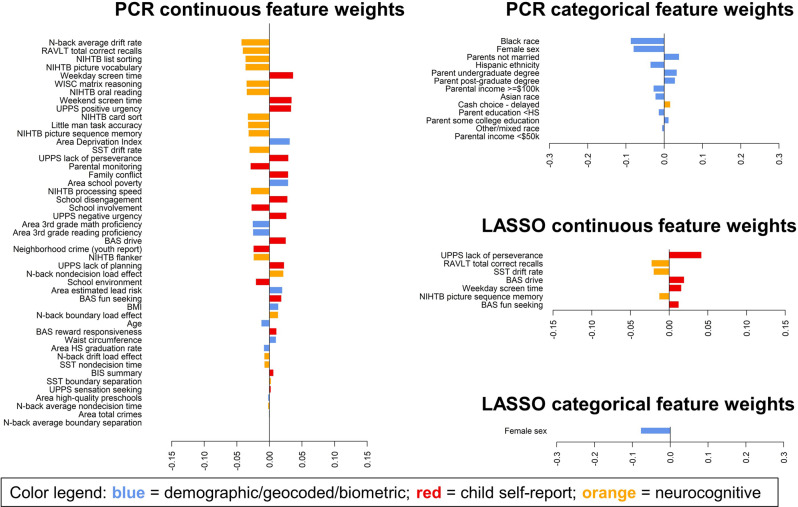
Feature weights for models predicting changes in ADHD symptoms from the baseline timepoint to the 1-year timepoint in the full sample with missing symptom data imputed. The bar plots display feature weights, averaged across training folds and ranked by absolute value, for the comprehensive PCR models and the sparse LASSO models. The sparse LASSO model only included eight of the most relevant features (i.e., those consistently selected in all training folds as well as both halves of the split-half analysis). Weights are color-coded by variable domain: blue = demographic/geocoded/biometric, red = child self-report, orange = neurocognitive testing. Continuous features are shown separately from categorical features because the predictor variables were scaled differently; continuous features were *z*-scored (mean = 0, SD = 1), while categorical features were coded as dummy variables with 0 = reference group versus 1 = feature present. RAVLT Rey Auditory Verbal Learning Test, NIHTB NIH Toolbox, WISC Wechsler Intelligence Scale for Children, SST Stop Signal Task, UPPS Impulsive Behavior Scale, BIS Behavioral Inhibition System, BAS Behavioral Approach System, HS High School, BMI body mass index.

Models predicting residual change scores were able to explain roughly 2% of the variance in symptom change between the baseline and 1-year time points (PCR *r* = 0.15, CI = 0.13–0.16; LASSO *r* = 0.15, CI = 0.13–0.17) and 1–2% of the variance in symptom change between the baseline and 2-year time points (PCR *r* = 0.12, CI = 0.09–0.15; LASSO *r* = 0.13, CI = 0.10–0.16). Prediction of change at the 1-year time point was mostly consistent across study sites, but the prediction of change at the 2-year time point was less consistent and was very poor for several sites (Fig. 1). As detailed above, the difficulty of predicting changes in symptoms was anticipated due to the well-known trait-like nature of ADHD symptoms and their high stability across waves of the study. Nonetheless, feature weights for predictors of 1-year symptom change (Fig. 4) and 2-year symptom change (Supplementary Fig. 7) showed a very similar pattern to feature weights predicting trait ADHD symptoms; cognitive measures, self-reported impulsivity, sex, and screen media use continued to be the most important predictors in these models.

## Discussion

We developed and tested machine learning models that use baseline neurocognitive, child self-report, and demographic features to predict children’s parent- and teacher-reported ADHD symptoms in the demographically diverse ABCD sample. We found that these models are highly generalizable across ABCD sites and robustly explain 12–20% of the variance in unseen data across several sensitivity analyses that varied in the time point at which ADHD symptoms were measured and the strategy used to address missing symptom data. Demographic, child self-report, and neurocognitive features all contributed meaningfully to prediction. Neurocognition accounted for roughly 4% of the variance beyond other domains and was featured heavily in a sparse predictive model that achieved comparable performance to more complex models.

Our findings are significant for addressing the longstanding debate on the clinical and practical utility of neurocognitive abilities for predicting ADHD symptoms [13, 16, 20–23]. When included in multivariate predictive models with diverse features from other domains, neurocognitive measures display clear added value. Although the results from the current study cannot be directly translated to clinical practice, they argue for the renewed consideration of neurocognitive measures in the clinical characterization of ADHD. The reliance of current clinical guidelines [60] on only a single measurement modality, informant rating scales, likely has significant drawbacks due to rater biases and effects of method variance [42] on measurement integrity (e.g., leading to inflated correlations with impairment measures drawn from the same modality and suppressed correlations with impairment measures from different modalities). Future work should assess whether neurocognitive measures can be used alongside informant scales or integrated with them in cross-modality measurement models (e.g., [61]) to improve measurement of the ADHD phenotype and its relations with impairment (especially impairment measures beyond informant ratings, such as grades and financial outcomes).

Although the magnitude of neurocognitive measures’ added value (*r*^*2*^ difference ~0.04) corresponds to a “moderate-sized” effect by common heuristics, comparison to empirical benchmarks (e.g., effects of anti-inflammatory and anti-histamine drugs, both of which are smaller) [62, 63] suggests it is non-trivial. Further, as the benefits of predictive informatics methods can be expected to cumulate (i.e., moderate improvements in prediction for individuals can translate to large overall impacts at the population level [62]), our findings suggest neurocognitive features could meaningfully improve the prediction of childhood ADHD symptoms at the level of large systems (e.g., national policies, healthcare enterprises) if not the level of individuals.

Relevant to this work, sparse models suggested that, in addition to a 6-test neurocognitive battery, the inclusion of only children’s sex, self-reported impulsivity, parental monitoring, and screen time was sufficient to achieve predictive accuracy comparable to models involving all 54 features investigated. The success of such a parsimonious model may prove particularly relevant for prediction in applied settings by reducing the number of measures needed.

Neurocognitive measures across many domains displayed negative relations with symptoms, consistent with recent evidence that neurocognitive development in youth largely reflects domain-general factors [64]. Computational model (DDM) parameters reflecting individuals’ accumulation of goal-relevant information across different tasks contributed significantly to prediction, while parameters reflecting other processes (e.g., n-back load effects, caution in responding) contributed little. This pattern is consistent with the hypothesis that domain-general efficiency of evidence accumulation is a key cognitive underpinning of ADHD [65]. However, as noted by a recent review of computational modeling applications to ADHD [12], most current tasks used in this literature are limited in their ability to allow for the accurate estimation of diverse model parameters due to having low numbers of trials and lacking experimental manipulations that are designed to modulate specific parameters. Hence, it is possible that novel tasks that allow for the estimation of a broader array of computational mechanisms (e.g., social cognition, reinforcement learning) could elucidate more specific cognitive predictors of ADHD symptoms.

Our findings notably contrast with two recent head-to-head comparisons of neurocognitive and survey-based measures that found neurocognitive measures contribute little to the prediction of ADHD [23] or related real-world outcomes [66]. A key reason for this discrepancy may be that the outcome variables in both studies were based on ratings by the same rater who completed self-report survey measures that were used as predictors, which allowed shared-method variance [42] to bias models toward surveys. The current study deliberately avoided this confound by excluding parent and teacher rating scales from the predictor matrix.

Beyond neurocognition, our analyses also highlight several other important predictors of childhood ADHD symptoms. Some are consistent with prior work, including lower parental monitoring [67] and self-reported impulsivity [68]. One of the most prominent and intriguing, however, is children’s estimates of their time spent engaging in screen media. Previous work in ABCD has found screen time to be associated with externalizing psychopathology, fluid cognitive ability, and neuroimaging markers [69, 70], but the current findings provide novel evidence that screen time is uniquely predictive of ADHD symptoms. As some of these important predictors, including both screen media use and parental monitoring, may be malleable or responsive to behavioral interventions, they represent potential targets for treatment. However, given that our prediction models do not necessarily reflect causal relationships, caution is warranted in this area.

Although models effectively predicted individuals’ overall level of ADHD symptoms at the 1-year and 2-year time points, predictions of symptom change from baseline to these time points were far less accurate. This was expected given prior research demonstrating that individuals’ ADHD symptoms are typically stable across development (especially the short intervals of 1–2 years available in the current study) [32–35], and the observation of strong correlations (*r* = 0.66–0.77) of the ADHD symptoms measure between time points. Predicting the difference between two highly correlated measures of the same trait is inherently challenging because much of the systematic variance in the individual measures is reflected in their correlation, and their difference will, therefore, necessarily contain a large portion of error variance relative to systematic variance [71, 72]. Indeed, the cross-timepoint correlations observed here are in the same range as test-retest reliability correlations reported for the rating scales from which the ADHD measure was derived [37, 38], suggesting differences in scores witnessed at 1- to 2-year intervals are largely due to error in the symptom measures themselves. Hence, for traits as stable as ADHD symptoms, changes across these intervals are unlikely to be meaningful. The residual change score analyses provide converging evidence for the importance of the features identified in the primary analyses predicting trait ADHD symptoms. However, rather than a prediction of “change over time”, these results may simply reflect the prediction of systematic variance in ADHD symptoms at the outcome timepoint that is present after residualizing for the imperfect baseline measure of the same trait.

It is also notable that overall predictive model performance was slightly worse in the full sample with missing ADHD symptom data imputed than in the smaller subsample with complete symptom data. This performance reduction could indicate an underestimation of model accuracy due to the imputation of teacher-report data with parent-report data, which are typically modestly related in a large portion of the sample or could reflect systematic differences between individuals with complete data and those with missing teacher ratings. Regardless, the similarity in contributions of predictive features and feature domains across these two analyses suggests that their role in predicting ADHD symptoms is robust to whether symptom data are imputed. Similarly, although predictions of the 2-year symptoms were less accurate than those of the 1-year symptoms, likely due to the greater temporal separation from baseline, the overall pattern of results suggests that ADHD symptoms represent a stable trait with relatively consistent predictors across time.

In summary, this study demonstrates that machine learning models can effectively utilize information about neurocognition and other youth characteristics, including sex, self-reported impulsivity, and screen time, to generate predictions about childhood ADHD symptoms that generalize to unseen data from diverse samples. This work provides a foundation for efforts to enhance the prediction of ADHD symptoms in ABCD and across broader research and clinical contexts.

## Supplementary information

SUPPLEMENTAL MATERIAL

## Data Availability

Data from the ABCD Study can be accessed through the NIMH Data Archive (NDA) repository detailed above in the Acknowledgements section and data specific to the current report can also be accessed in a separate NDA Study (10.15154/3x5k-xa83).
