# Supplementary material for: Generalizable prediction of childhood ADHD symptoms from neurocognitive testing and youth characteristics
Source: Transl Psychiatry. 2023 Jun 24;13:225. doi: 10.1038/s41398-023-02502-6 (PMC10290685; doi:10.1038/s41398-023-02502-6)
Supplement: Supplementary file 1 — SUPPLEMENTAL MATERIAL [file 41398_2023_2502_MOESM1_ESM.docx]

**Supplemental Material for:** Generalizable prediction of childhood ADHD symptoms from neurocognitive testing and youth characteristics

Alexander Weigard, Ph.D.^1^, Katherine L. McCurry, Ph.D.^1^, Zvi Shapiro, Ph.D.^2^, Meghan E. Martz, Ph.D.^1^, Mike Angstadt, M.S.^1^, Mary M. Heitzeg, Ph.D.^1^, Ivo D. Dinov, Ph.D.^3^ & Chandra Sripada, MD, Ph.D.^1^

^1^Department of Psychiatry, University of Michigan

^2^Department of Psychology, Emory University

^3^Departments of Computational Medicine and Bioinformatics, and Health Behavior and Biological Sciences, University of Michigan

**Cross-Rater ADHD Symptoms Measurement Model**

We aimed to create an outcome measure of ADHD symptoms that i) was informed by children’s behavior across the home and school contexts and ii) was as free of rater-specific biases (e.g., parental distress, teacher associations of behavior with academic performance) as possible. Bifactor models specified with structural equation modeling provide valuable tools for separating variance in a general factor common across measurement sources from source-specific variance^1^. We, therefore, developed a bifactor measurement model that estimated a general, cross-rater ADHD symptoms factor informed by both parent Child Behavior Checklist (CBCL) ratings and teacher Brief Problem Monitor (BPM) ratings as well as orthogonal parent-specific and teacher-specific subfactors.

Initial model development work was conducted with a subsample of 4,258 individuals who had full parent- and teacher-report data, who were not from the three “lockbox” sites, and who were unrelated (one individual from each family was randomly selected for inclusion to prevent family-related clustering from biasing the standard errors of parameter estimates). We used the *lavaan*^2^ R package to fit all structural equation models and to generate cross-rater ADHD symptoms factor scores for the subsequent predictive modeling analyses. As the individual CBCL and BPM items are ordinal, rather than numeric, variables, we used the WLSMV estimator to fit all structural equation models.

We began by estimating an “unconstrained” bifactor model in which all 14 CBCL items that are included in either the Attention Problems syndrome scale or the ADHD DSM-oriented scale were allowed to load on the parent-specifics subfactor, the 6 BPM items of the Attention/Hyperactivity Problems subscale were allowed to load on the teacher-specific subfactor, and all 20 of these items were allowed to load on the general, cross-rater factor. The resulting model displayed good fit to the data according to standardized fit indices (RMSEA = 0.025, SRMR = 0.039, CFI = 0.992, TLI = 0.989) and factor loadings that were uniformly statistically significant (*p*<0.001, likely due to the large sample size).

However, an inspection of the standardized loadings (Supplemental Table 1) indicated that several were relatively weak (<0.30). Specifically, four items that are included in the CBCL Attention syndrome scale but are not typically considered symptoms of ADHD (“Confused or seems to be in a fog,” “Nervous, highstrung, or tense,” “Poorly coordinated or clumsy,” and “Stares blankly”), loaded weakly on the general factor. In addition, two CBCL items that loaded strongly on the general factor loaded weakly on the parent-specific subfactor (“Acts too young for his/her age” and “Poor school work”). The fact that all four items with weak loadings on the general factor displayed poor face validly as measures of ADHD symptoms suggested that their general factor loadings could be removed in order to create a more parsimonious model that would be better constrained by the data. The two CBCL items with weak loadings on the parent-specific subfactor appeared to be good indicators of children’s ADHD symptoms but poorer indicators of parent-specific biases that are, in theory, reflected by the subfactor (e.g., parental distress about psychopathology symptoms). This similarly suggested that these two loadings could be removed to better constrain the model. Better-constrained models tend to be easier to estimate and to have more stable parameter values, both of which were valuable features in the context of the present study, given that the ADHD symptoms measurement model needed to be re-fit separately to all training folds in cross-validation analyses. We, therefore, estimated a more constrained model that eliminated all six of these weak factor loading parameters.

The constrained model displayed standardized fit indices that were worse than those of the unconstrained model (as expected for any model with fewer free parameters) but were still generally within acceptable ranges (RMSEA = 0.062, SRMR = 0.080, CFI = 0.949, TLI = 0.937). The factor loadings that remained in the constrained model were relatively unchanged from their corresponding loading values in the unconstrained model (Supplemental Table 1). Furthermore, factor scores for the cross-rater ADHD symptoms factor were nearly perfectly correlated between the constrained and unconstrained model versions (*r*=0.98), suggesting that measurement of the outcome of interest was highly robust to the constraints we imposed. We, therefore, decided to use the constrained model to measure cross-rater ADHD symptoms in all prediction analyses.

Although the parameters of the measurement model had to be re-estimated within each training fold to prevent data bleed between the training and test data, we anticipated that, given the large sample size, the resulting factor scores for cross-rater ADHD symptoms would be highly similar between the models from each fold. Indeed, when factor scores were generated for all participants from the models separately estimated within each cross-validation training fold, these factor scores were all nearly perfectly correlated with one another (all *r*s>0.99). Hence, we can be confident that any differences in the ADHD symptoms outcome measure between the cross-validation folds were trivial.

We then sought to assess the temporal stability of the cross-rater ADHD symptoms factor. We first fit the constrained measurement model to a subsample of ABCD baseline data from 2,855 unrelated individuals who had complete parent- and teacher-report data and who were not in the “lockbox” sites. For the individuals in the intersection of our baseline and year-1 analytic subsamples (*n*=1,566), the cross-rater ADHD symptoms factor displayed a correlation coefficient of *r* = 0.77 across the two timepoints, suggesting that this factor showed high temporal stability across the 1-year measurement interval. Next, using the multiple imputation procedures described in the section below, we fit the measurement model to a larger set of unrelated individuals with missing data imputed for the baseline time point (*n* = 7,866) and then to the same subset of individuals who also had data available at the 1-year (*n* = 7,401), and 2-year (*n* = 6,783) time points. The correlation between the baseline and 1-year scores was *r* = 0.71, and the correlation between the baseline and 2-year scores was *r* = 0.66. Although slightly lower than the temporal stability estimate in the smaller sample with complete data, these correlations suggest that the rank-ordering of individuals’ ADHD symptoms is relatively temporally stable across both the 1- and 2-year intervals.

**Imputation of ADHD Symptom Data and Sensitivity Analyses**

Missing teacher-report data, and a smaller portion of the parent-report data for individuals who were missing items from the CBCL, were imputed using multivariate imputation using chained equations, as implemented by the *mice* R package^3^. To prevent data leakage between the outcome and predictor variables, only items from the CBCL and BPM were entered into multiple imputation analyses for ADHD symptom items (i.e, no predictor variables were entered). To further prevent information leakage between training and test data, the imputation procedure was completed separately with ADHD symptom data from each training fold, and the same imputation model derived from a given training fold was used to impute missing ADHD symptom data in the corresponding left-out test site with the *mice.reuse()* function (github.com/prockenschaub/Misc/tree/master/R/mice.reuse).

Given that parent-report data on ADHD symptoms typically show modest relations with teacher-report data^4^, we were initially concerned that imputing teacher-report data using only parent-report data would lead to poor estimates of cross-rater ADHD symptoms and, in turn, underestimation of predictive model performance. Therefore, we completed several sensitivity analyses to gauge the impact of imputing teacher-report data on the outcome measure.

We first assessed whether imputation substantially altered the factor loadings and cross-rater ADHD symptoms factor scores from the constrained measurement model. When the model was fit to the subsets of unrelated individuals included in the temporal stability analyses described above from the 1-year (*n* = 7,401) and 2-year (*n* = 6,783) time points, 3,341 (45%) and 4,041 (59%) of participants had their teacher-report data imputed, respectively. We compared the factor loadings and scores from these model fits to those obtained from the model fit to only individuals with complete data at the 1-year time point. The constrained model displayed acceptable fit to both the 1-year (RMSEA = 0.059, SRMR = 0.075, CFI = 0.948, TLI = 0.937) and 2-year (RMSEA = 0.051, SRMR = 0.066, CFI = 0.955, TLI = 0.945) samples with imputed data. Despite some slight reductions in the standardized loadings of teacher-report items on the cross-rater ADHD symptoms factor, the general pattern of loadings in these samples with imputed data (Supplemental Table 2) was highly similar to that from the original analysis of individuals with complete data (Supplemental Table 1). Moreover, when factor scores from the original model fit to complete 1-year data were compared with factor scores from the models fit to the samples with imputed data, they were nearly perfectly correlated (both *r*s>0.99). This set of analyses demonstrates that the measurement model and factor scores for the cross-rater ADHD symptom measure of interest are robust to whether multiple imputation is used to replace missing teacher-report data.

We also conducted an analysis explicitly aimed at determining how closely factor scores generated with imputed teacher-report data correspond to factor scores generated when empirical teacher-report data are present. To do so, we focused on the sample of all available individuals who were not part of the lockbox sites and had any ADHD symptom data available at the 1-year time point (*n* = 8,972). We randomly selected 1,000 of these individuals who had full empirical teacher-report data and fit the constrained measurement model to the data set following multiple imputation in two conditions; when the empirical teacher-report data for these 1,000 individuals were present versus when these data were artificially deleted and had to be imputed. We then compared the cross-rater ADHD symptoms factor scores for these 1,000 participants when their data were present to when the data were imputed. The scatterplot of these values indicated a close correspondence between scores from the two analyses (Supplemental Figure 1), and the factor scores were correlated at *r* = 0.96, indicating about 92% of the variance was shared between the scores obtained from complete versus missing teacher-report data. Therefore, to our surprise, the cross-rater ADHD symptoms factor scores appear to be reliably estimated even for individuals with missing teacher-report data. However, the slight differences between scores estimated from complete teacher-report data versus imputed data may nonetheless explain some of the reductions in predictive model performance for the larger samples with imputed data versus the 1-year sample of individuals with complete teacher-report data, as reported in the main manuscript.

**Demographic, Geocoded, and Biometric Features**

Descriptive statistics and proportions of missing data for all demographic, geocoded, and biometric variables are displayed in Supplemental Table 2.

*Basic demographic features* included age at baseline, in months, and several categorical variables, the levels of which were determined using the procedures outlined in the scripts shared by the ABCD Data Analytics and Informatics Resource Center (github.com/ABCD-STUDY/analysis-nda). Male sex was used as the reference level for sex (Male/Female) in all regression-based analyses. Race was split into White, Black, Asian and Other/Mixed categories, with White being used as the reference level. Ethnicity was split into Hispanic versus non-Hispanic categories, with the latter being used as the reference level. Parent marital status was split into Married versus Unmarried categories, with the former serving as the reference level. Parental income was split into <$50k annually, >$100k annually, and a middle category between these two that served as the reference level. Highest parental education was split into “< High School,” “High School / GED,” “Some College” (i.e., less than a 4-year degree), “Bachelor,” and “Post-Graduate” (any graduate school). Due to the relatively low number of individuals in the “< High School” group, which is often used as a reference group in regression analyses, we instead used the “High School / GED” group as a reference (although we continued to estimate effects of being in the “< High School” group compared to this reference).

*Geocoded features* were created by the ABCD Data Analysis Informatics and Resource Center (DAIRC) by determining the longitude and latitude of each participant’s primary residential address and matching this location with available external data sources aggregated at different geospatial resolutions (e.g., state or U.S. Census tract), as described in detail elsewhere^5^. We included an aggregate measure of neighborhood poverty composed of 9 items from the Area Deprivation Index (derived at the level of U.S. Census tract from the American Community Survey) that was recently developed for use in ABCD and linked to several cognitive and neuroimaging indices^6^. The aggregate measure was computed by taking the average of these items’ standardized *z*-scores (mean = 0; SD =1). To preserve independence of training and test data, we re-calculated *z*-scores in each fold of the training data prior to fitting predictive models and then applied the same scaling (i.e., same assumed means and SDs for each variable) to the respective test data before testing the models. We also included measures of the total number of crimes reported at the county level, risk for lead exposure (imputed based on age of homes and poverty level for children’s Census tract), and several indices of education system quality and educational opportunity in the area: high school graduation rate, 3^rd^ grade reading and math proficiency, school poverty, and availability of high-quality preschools.

*Biometric features* included children’s waist circumference (in inches) and body mass index (BMI), calculated from data collected using the National Health and Nutrition Examination Survey. We found that a small number (<0.1%) of computed BMI values were implausible (>100 or <10), likely due to erroneous measurement of children’s height and/or weight. These values were trimmed and treated as missing data.

**Child Self-Report Features**

Given the association of ADHD symptoms with impulsivity and reward-seeking behavior, children’s self-reports on two measures of these and related personality traits were used as predictive features (Supplemental Table 3). The Behavioral Inhibition System / Behavioral Activation System (BIS/BAS) scale^7^ contains three subscales thought to measure traits associated with reward-seeking and positive affect (BAS: Reward Responsiveness, Drive, and Fun Seeking) and a single subscale measuring behavioral inhibition (BIS Summary), which is thought to represent sensitivity to punishment. A short form of the Urgency, Premeditation (lack of), Perseverance (lack of), Sensation Seeking, Positive Urgency, Impulsive Behavior Scale (UPPS-P) developed for use in ABCD^8^ contains 5 subscales: Negative Urgency, Positive Urgency, Lack of Perseverance, Lack of Planning, and Sensation Seeking.

Children’s perceived level of family conflict was measured by the Family Conflict subscale of the Family Environment scale^9^ in the ABCD PhenX Toolkit^10,11^. Youth reported on their perceived level of parental monitoring, the degree to which their parents keep track of their whereabouts and activities within and outside the home^12^, on a 5-point PhenX scale. Neighborhood crime/safety was also evaluated on a single 5-point scale (“My neighborhood is safe from crime”). Children’s perception of the school environment and their engagement in school was evaluated with a 12-item measure from the PhenX Toolkit that yielded the following three subscales: a 6-item School Environment scale, a 4-item School Involvement scale and a 2-item School Disengagement scale.

Children reported the amount of time on a typical weekday and weekend day that they spent engaged in 6 types of screen media: watching TV shows or movies, watching videos (e.g., on YouTube), playing video games, texting, visiting social networking sites, and video chatting. The response options were coded as: 0 = “None”, .25 = “< 30 minutes”, 0.5 = “30 minutes”, 1 = “1 hour”, 2 = “2 hours”, 3 = “3 hours”, and 4 = “4+ hours”. For weekdays and weekend days separately, we summed across responses for all 6 categories to obtain summary measures of screen time.

**Neurocognitive Features: ABCD Cognitive Battery**

We included all measures administered as part of the ABCD cognitive battery^13^ at the behavioral testing session. This battery included the complete set of NIH Toolbox measures as well as a handful of other tests that were selected to extend the cognitive domains assessed.

The NIH Toolbox is a computerized battery of 7 tests designed to span the cognitive domains of episodic memory, working memory, attention, processing speed, and verbal ability^14^. We included age-corrected scores from all 7 tests, which are described briefly here and in greater detail elsewhere^13,14^, as features in our predictive models. The Picture Vocabulary test is a variant of the Peabody Picture Vocabulary Test (PPVT) in which children are presented with auditory vocabulary words and must select one of four presented pictures that best represents each word. The Oral Reading test involves asking children to pronounce single letters and words that are presented visually on a tablet. The Pattern Comparison Processing Speed test presents two pictures on each trial and requires that participants respond as quickly as possible, via touch input, as to whether or not the pictures are the same. The List Sorting Working Memory test involves the serial presentation of pictures and auditory names of animals or foods of different sizes followed by a request for the participant to repeat back the items that were presented in a given series in the order of smallest to largest size. The Picture Sequence Memory test asks participants to reproduce a sequence of events presented in a series of 15 pictures depicting activities that occur in a particular setting (e.g., working on a farm). The Flanker test is a variant of the traditional arrow flanker paradigm^15^ in which children are presented with trials in which the four flanking arrows are either facing the same direction (congruent trials) or the opposite direction (incongruent trials) as a central target arrow. The Dimensional Change Card Sort test is thought to measure cognitive flexibility by asking children to sort objects according to two different dimensional criteria (color and shape) that alternate across blocks and that are interleaved pseudo-randomly in a third block.

Several additional measures beyond the NIH Toolbox were also administered. The Rey Auditory Verbal Learning Test (RAVLT)^16^ is a learning and memory measure in which participants are presented with a list of 15 unrelated words across 5 learning trials, are subsequently presented with a distractor list of 15 different words, and are then asked to recall the original list, both immediately and following a 30-minute interval. As we found that individuals’ total correct recalls were strongly correlated across all trials (typically >0.70), suggesting that much of the variance in scores could be attributed to a single cognitive dimension, we summed total correct responses across all trials to create a single feature for our predictive models. The Matrix Reasoning subtest of the Wechsler Intelligence Scales for Children (WISC-V)^17^ is a commonly used measure of general intelligence and fluid reasoning ability in which, for each trial, participants are shown a series of visuospatial stimuli that represents a logical pattern and must select which one of four additional stimuli complete the pattern. We used age-adjusted “standard scores” (mean=10, SD=3) derived from this measure as a predictive feature. The Little Man Task^18^ is a measure of visuospatial processing in which participants are shown a male stick figure holding a briefcase in either his left or right hand that is presented in the middle of the screen in one of several positions: either right side up or upside down and either facing the participant or with his back to the participant. The participant is asked to respond as to which hand the man is holding the briefcase with. We used overall accuracy rate from the Little Man Task as a predictive feature. Finally, the Cash Choice Task was designed to measure delay of gratification by asking participants to answer the following question: “Let’s pretend a kind person wanted to give you some money. Would you rather have $75 in three days or $115 in 3 months?”.

**Neurocognitive Features: Diffusion Model Parameters from Neuroimaging Tasks**

The diffusion decision model (DDM) is a formal computational model that explains response time (RT) and accuracy data across a wide variety of cognitive paradigms as resulting from a process in which individuals gradually accumulate noisy evidence for each possible choice until the evidence total crosses a critical boundary for one of the choices^19,20^. The DDM has been widely applied to empirical data, typically with the goal of estimating several of its parameters that are thought to reflect key mechanisms of cognitive processing. These include the “drift rate” (*v*) parameter, which represents the efficiency with which individuals selectively gather evidence for adaptive choices, the “boundary separation” (*a*), which represents the amount of evidence an individual requires before finalizing a choice, and the “nondecision time” (*t0*), which accounts for the timing of perceptual and motor operations that occur either before or after the choice-selection process. In individual differences research, these parameters are commonly estimated as measures of individuals’ cognitive efficiency (*v*), caution in responding (*a*), and peripheral/perceptual motor processes (*t0*). In addition, the DDM contains a “start point” parameter that accounts for biases towards one of the two responses (*z*). Parameters for between-trial variability in drift rate (*sv*), start point (*sz*), and nondecision time (*st0*) are also included in the full model, although of these only the *st0* parameter can be accurately estimated without large amounts of RT data at the individual level^21^.

Two of the tasks completed during the ABCD neuroimaging session^22^ meet the DDM’s general assumptions and have also both been previously used to estimate parameters from the DDM, and related models, in prior work: the stop signal task (SST)^23,24^ and N-back task^25,26^. In the SST, participants are presented with right- or left-facing arrows and must respond as to the direction of the arrow. On a subset of “stop” trials (60 out of 360 total trials), participants are presented with an upward-facing arrow shortly after presentation of the initial choice stimulus and must inhibit their response. Following previous model applications to the SST^23,24^, we used choice RT data from the 300 “go” trials to estimate DDM parameters. The N-back task was an “emotional” variant of the standard paradigm in which participants were displayed serial arrays of either face images (expressing different emotions) or building images. As is typical of n-back paradigms, the task contains different levels of working memory load. In the 0-back (“low” load) condition, participants were shown a picture of a “target” image at the beginning of each block of 10 trials and were asked to respond as to whether each subsequent image in the block was the target image or not. In the 2-back (“high” load) condition, participants were asked to respond as to whether each stimulus in a 10-trial block was the same as the stimulus presented two spaces back in the series (i.e., the “target” image is always the same as the image presented two spaces earlier). Participants completed 8 blocks in each load condition, making for a total of 80 0-back and 2-back trials. At both levels of load, stimuli on a given trial could be: 1) target images, 2) “non-target” images, which are images that were never presented before, or 3) “lure” images, which were previously presented in the block but are not targets. Lures are more difficult to reject than non-targets, as they are familiar to participants, and are typically included in n-back designs to ensure that participants must actively maintain target-relevant information rather than relying solely on familiarity^27^.

For the SST, we estimated a single DDM that explained data across all “go” trials using the core model parameters (*v*, *a*, *t0*, *z*) and the nondecision time variability parameter (*st0*). We did not estimate the other variability parameters (*sz*, *sv*) due to the difficulties of accurately doing so without large numbers of trials^21^. For the n-back, we fit the DDM separately to the 0-back and 2-back tasks. These DDMs estimated single *a*, *t0*, *z*, and *st0* parameters for each load level and allowed the *v* parameter to vary across target, non-target, and lure trials, leading to three separate *v* estimates at each load level. Following recent work on omissions^28^, we accounted for non-responding in all tasks by adjusting the likelihood function for the proportion of RTs that would be expected to occur after the response window as well as by including a separate parameter for the probability of “contaminant” omissions (e.g., due to distraction).

The DDM was fit to trial-level RT data from all tasks using Bayesian estimation with the differential evolution Markov chain Monte Carlo (DE-MCMC)^29^ method in the Dynamic Models of Choice (DMC)^30^ R suite. Prior to estimation, we excluded individuals’ data for a given task if they displayed accuracy rates close to chance (<55%) or omission rates greater than 25%. We also excluded trials with RTs less than 200ms as likely fast guesses. We created informative priors for DDM parameters using a framework we previously developed^31^ in which a hierarchical version of the cognitive model is fit to a 300-person subsample that is independent of the participants included in the main analyses and is then used to generate priors for individual-level fits to rest of the sample. This strategy can provide helpful constraints on individuals’ parameter estimates without the drawbacks of using a fully hierarchical approach with all participants (e.g., the considerable demands on computing resources and the non-independence of individual-level estimates from hierarchical models). The 300 participants for the independent prior-generation subsample in the current study were randomly drawn from the pool of ABCD participants who: 1) were not from one of the three “lockbox” sites, 2) did not have siblings in the sample, 3) did not have complete parent- and teacher-report data at any of the baseline, 1-year, or 2-year timepoints (this last criterion was to make sure sensitivity analyses of the subset of individuals with complete ADHD symptom data were maximally powered). After estimating hierarchical DDMs for each task with data from this 300-person subsample, we fit truncated normal distributions to the full distribution of all individual-level posterior samples for the DDM parameters from each hierarchical model. The resulting truncated normal distributions were then used as informative priors for the individual-level Bayesian estimation of DDM parameters for participants included in our main analyses. Code for all modeling procedures is available on this study’s OSF page.

Model fit was evaluated with posterior predictive plots^32^ that compared, for three randomly selected ABCD sites, the average empirical probabilities and latencies of each type of response with the corresponding probabilities and latencies predicted by the DDM (Supplemental Figures 2-4). Inspection of these plots indicates that the DDM provides an excellent description of the empirical data, with the greatest misfit to lure trials in the 2-back task, although this misfit is relatively minor. Parameter recovery studies with simulated data that replicated the trial numbers and observed parameter ranges of the ABCD data suggested that the main parameters of interest (*v*, *a*, *t0*) were all recovered well from the SST task (*r* between simulated and recovered parameter values > 0.75). The *v* and *t0* parameters were recovered similarly well for the 0-back and 2-back tasks (*r* > 0.75), and the *a* parameter displayed slightly poorer recovery (*r* ~ 0.59-0.62).

Point estimates of the three main parameters of the DDM (*v*, *a*, *t0*) were obtained by taking the median of individuals’ posterior samples for each parameter. Predictive analyses included: 1) the three main DDM parameters estimated from the SST, 2) the average of each of these DDM parameters across targets, non-targets and lures and across both load levels in the n-back, and 3) n-back working memory load effects (0-back minus 2-back) in each of the three parameters.

**Model Training, Testing, and Variable Importance Weights**

Prior to training predictive models, missing data for all predictor features were imputed with multivariate imputation using chained equations, as implemented by the *mice* R package^3^. The criterion variable (ADHD symptoms) was left out of all imputation analyses to prevent information leakage from contaminating the imputed predictors. To further prevent information leakage between the training and test data, the imputation procedure was completed separately within each training fold, and the same imputation model derived from a given training fold was used to impute missing data in the corresponding left-out test site with the *mice.reuse()* function (github.com/prockenschaub/Misc/tree/master/R/mice.reuse). Visual inspections of plots comparing observed and missing data for selected features (among those with the greatest proportion missing) were used to assess the effectiveness of the imputation procedure, including univariate histograms, bivariate relationship plots, and plots of the relation between each feature and its missingness propensity score. Inspection of these plots suggested that the multiple imputation procedure sufficiently replicated the distributional properties and key bivariate relationships of features with nontrivial amounts of missing data. For analyses in which data from different measurement domains were alternately left in versus out of the predictive model (e.g., using cognitive data alone, using all data except child self-report, etc.), the multiple imputation procedure was repeated using only the data that were left in each analysis. This procedure prevents leakage from domains outside of the left-in data from inadvertently contributing to prediction by informing missing data imputations.

Following imputation, continuous features in each training fold were standardized using *z*-scores (mean = 0; SD =1) to improve interpretability of model parameters. To ensure that the test data were on the same scale as the training data, the means and SDs of continuous measures in the training fold were also used to scale data in the corresponding left-out test site. Categorical training and test data were dummy coded by creating separate variables for each level relative to a reference level (1 = feature present, 0 = absent).

*Principal components regression* (PCR) was implemented using principal components analysis (PCA) functions from the *FactoMineR*^33^ R package and custom R functions available on the current study’s OSF page. To train the model, a PCA was first conducted with all continuous features (categorical features are inappropriate for PCA and were therefore left out of this step) and used to generate component scores for all principal components. Next, a 10-fold “inner loop” cross-validation procedure was implemented within the training data to identify the optimal number of components to include in the predictive model. For each of the 10 inner loop training folds and each possible number of components (one component minimum and the maximum number of components being one less than the number of continuous features), scaled component scores were entered along with categorical features into a regression model predicting ADHD symptoms. The resulting models were used to predict ADHD symptoms in the 1/10^th^ of the training data that was left out, and the correlation coefficient (*r*) for the relation between the predicted and observed ADHD symptoms scores was averaged for each number of components across all 10 left-out folds. The number of components that achieved the highest average *r* value (rounded to two decimal places to prevent trivial differences in prediction from influencing the number of components selected) was retained as the “winning” model. The model with the winning number of components was then estimated in the full training data to establish the final PCR predictive model for that training fold. To test this PCR model, the PCA from the training data was used to generate component scores in the left-out test site (which were scaled according to the means and SDs of component scores from the training data to ensure identical scaling). The resulting scaled component scores were entered along with categorical variables into the trained PCR model to predict ADHD symptoms for left-out participants.

*Least absolute shrinkage and selection operator regression* (LASSO) was implemented using functions from the *glmnet* R package^34^. The regularization parameter λ was selected separately for each training fold using an inner loop cross-validation procedure similar to the above procedure for selecting the number of components in PCR models. Specifically, the *cv.glmnet()* function was used to conduct 10-fold cross-validation within each training fold, and the largest λ value within one standard error of the minimum mean squared error for cross-validated predictions was selected. We found that this λ value worked well for the primary analyses predicting individuals’ trait ADHD symptoms but was frequently too conservative for analyses predicting change scores, often leading all predictors to be left out of the model due to their relatively weak associations with the change scores. Therefore, for the change score analyses, we adopted the more liberal λ value that corresponded to the minimum mean squared error. These LASSO models were fit to the full training data set and were then used to generate predicted ADHD symptoms scores for left-out participants.

To evaluate whether LASSO could be used to generate a sparse model that achieves optimal performance with only a small set of variables, we conducted additional analyses in which we established a stringent threshold for variable inclusion. We only included variables selected by LASSO (i.e., had non-0 parameter estimates) in all 18 training folds within the main analyses. As the data in these folds were mostly overlapping, we also conducted a split-half analysis in which LASSO was fit separately to two independent subsamples made up of 9 randomly selected ABCD sites each. Only variables present at the intersection of the LASSO models from both halves and the set of variables that were included across all 18 training folds were determined to be robust enough for inclusion in the final sparse LASSO model. After the multiple imputation step was carried out with only these robust variables included in the data set, we conducted the same LASSO training and testing procedures described above with leave-one-site-out cross-validation across all 18 sites.

Feature weights for continuous variables from PCR were computed by multiplying the matrix of features’ standardized component loadings by the matrix of beta coefficients from each trained regression model, which are comparable to standardized beta coefficients because the components were standardized prior to entry in the regression. Feature weights for categorical variables in PCR were simply their beta coefficients from the final model and were considered separately from the continuous feature weights due to their scaling differences with continuous variables (0/1 dummy coding versus *z*-scores). Feature weights for LASSO were simply coefficients obtained from the final sparse LASSO model. For both PCR and LASSO, the feature weights reported in Figure 2 were generated by averaging weights across all 18 folds of the leave-one-site-out cross-validation procedure.

**Procedures for Predicting Residual Change Scores**

The same general machine learning and cross-validation procedures that were used to predict trait ADHD symptoms at the 1-year and 2-year time points, as described above and in the main body of the mansucript, were also used to predict the residual change scores (Δ) for changes in symptoms from baseline to these time points, with two exceptions.

Firstly, the Δ outcome variables were generated separately in each training fold to prevent data leakage in cross-validation. This process involved estimating the ADHD symptom measurement model with data from the outcome time point (1-year or 2-year), using this estimated model to obtain cross-rater ADHD symptom factor scores for both the outcome timepoint and the baseline timepoint, regressing the outcome factor scores on the baseline factor scores, and generating residual change scores from this regression. After completing this process with the training data, the same estimated measurement model was used to generate cross-rater ADHD symptom factor scores for individuals in the test site at both time points, and the same regression model was used to generate the Δ outcome scores in the test site.

Secondly, we used more liberal values of the LASSO regularization parameter, λ, which is determined via an “inner loop” cross-validation procedure (see Supplemental Materials). Although, for the main analyses predicting trait ADHD symptoms, we used largest λ value within one standard error of the minimum mean squared error for cross-validated prediction, use of this λ value when predicting change scores often led to all variables being excluded from the model due to the much weaker associations between predictor features and change scores. Therefore, for the Δ prediction analyses, we instead adopted the λ value that led to the minimum mean squared error, which is more liberal in its inclusion of predictor variables in the model.

**Supplemental Tables and Figures**

**Supplemental Table 1**. Standardized parameter estimates for all factor loadings of parent Child Behavior Checklist (CBCL) and teacher Brief Problem Monitor (BPM) items across the constrained and unconstrained bifactor models.

|  | **Unconstrained Model** | | | **Constrained Model** | | |
| --- | --- | --- | --- | --- | --- | --- |
| **Scale/Item** | **General factor loading** | **Parent-specific loading** | **Teacher-specific loading** | **General factor loading** | **Parent-specific loading** | **Teacher-specific loading** |
| BPM - Acts too young for his/her age | 0.41 |  | 0.42 | 0.42 |  | 0.42 |
| BPM - Fails to finish things he/she starts | 0.52 |  | 0.47 | 0.52 |  | 0.47 |
| BPM - Can't concentrate, can't pay attention for long | 0.58 |  | 0.67 | 0.58 |  | 0.67 |
| BPM - Can't sit still, restless, or hyperactive | 0.42 |  | 0.59 | 0.43 |  | 0.59 |
| BPM - Impulsive or acts without thinking | 0.46 |  | 0.54 | 0.46 |  | 0.54 |
| BPM - Inattentive or easily distracted | 0.55 |  | 0.70 | 0.56 |  | 0.69 |
| CBCL - Fails to finish things he/she starts | 0.54 | 0.38 |  | 0.52 | 0.43 |  |
| CBCL - Can't concentrate, can't pay attention for long | 0.72 | 0.38 |  | 0.68 | 0.49 |  |
| CBCL - Can't sit still, restless, or hyperactive | 0.63 | 0.33 |  | 0.60 | 0.43 |  |
| CBCL - Impulsive or acts without thinking | 0.55 | 0.37 |  | 0.52 | 0.43 |  |
| CBCL - Inattentive or easily distracted | 0.69 | 0.44 |  | 0.67 | 0.53 |  |
| CBCL - Talks too much | 0.36 | 0.32 |  | 0.35 | 0.33 |  |
| CBCL - Unusually loud | 0.34 | 0.36 |  | 0.34 | 0.36 |  |
| CBCL - Acts too young for his/her age | 0.44 | 0.29 |  | 0.55 |  |  |
| CBCL - Confused or seems to be in a fog | 0.27 | 0.39 |  |  | 0.58 |  |
| CBCL - Daydreams or gets lost in his/her thoughts | 0.34 | 0.42 |  | 0.33 | 0.42 |  |
| CBCL - Nervous, highstrung, or tense | 0.22 | 0.46 |  |  | 0.58 |  |
| CBCL - Poor school work | 0.60 | 0.10 |  | 0.63 |  |  |
| CBCL - Poorly coordinated or clumsy | 0.29 | 0.38 |  |  | 0.59 |  |
| CBCL - Stares blankly | 0.28 | 0.43 |  |  | 0.64 |  |

**Supplemental Table 2**. Standardized parameter estimates for all factor loadings of parent Child Behavior Checklist (CBCL) and teacher Brief Problem Monitor (BPM) items when the constrained bifactor measurement model is fit to a subset of unrelated individuals from the 1-year (*n* = 7,401) and 2-year (*n* = 6,783) time points who had missing symptom report data imputed if needed.

|  | **1-Year Data** | | | **2-Year Data** | | |
| --- | --- | --- | --- | --- | --- | --- |
| **Scale/Item** | **General factor loading** | **Parent-specific loading** | **Teacher-specific loading** | **General factor loading** | **Parent-specific loading** | **Teacher-specific loading** |
| BPM - Acts too young for his/her age | 0.36 |  | 0.42 | 0.30 |  | 0.45 |
| BPM - Fails to finish things he/she starts | 0.49 |  | 0.50 | 0.38 |  | 0.56 |
| BPM - Can't concentrate, can't pay attention for long | 0.54 |  | 0.70 | 0.42 |  | 0.76 |
| BPM - Can't sit still, restless, or hyperactive | 0.38 |  | 0.59 | 0.33 |  | 0.60 |
| BPM - Impulsive or acts without thinking | 0.40 |  | 0.55 | 0.33 |  | 0.56 |
| BPM - Inattentive or easily distracted | 0.52 |  | 0.71 | 0.43 |  | 0.79 |
| CBCL - Fails to finish things he/she starts | 0.53 | 0.43 |  | 0.53 | 0.44 |  |
| CBCL - Can't concentrate, can't pay attention for long | 0.68 | 0.49 |  | 0.68 | 0.48 |  |
| CBCL - Can't sit still, restless, or hyperactive | 0.59 | 0.42 |  | 0.56 | 0.41 |  |
| CBCL - Impulsive or acts without thinking | 0.52 | 0.41 |  | 0.53 | 0.43 |  |
| CBCL - Inattentive or easily distracted | 0.66 | 0.53 |  | 0.65 | 0.53 |  |
| CBCL - Talks too much | 0.35 | 0.34 |  | 0.29 | 0.34 |  |
| CBCL - Unusually loud | 0.33 | 0.35 |  | 0.32 | 0.32 |  |
| CBCL - Acts too young for his/her age | 0.55 |  |  | 0.54 |  |  |
| CBCL - Confused or seems to be in a fog |  | 0.59 |  |  | 0.56 |  |
| CBCL - Daydreams or gets lost in his/her thoughts | 0.32 | 0.42 |  | 0.27 | 0.47 |  |
| CBCL - Nervous, highstrung, or tense |  | 0.56 |  |  | 0.54 |  |
| CBCL - Poor school work | 0.63 |  |  | 0.66 |  |  |
| CBCL - Poorly coordinated or clumsy |  | 0.57 |  |  | 0.57 |  |
| CBCL - Stares blankly |  | 0.62 |  |  | 0.57 |  |

**Supplemental Table 3**. Descriptive statistics and missing data proportions for demographic, geocoded, and biometric variables used as features in the predictive models. Values are from all individuals who met basic inclusion criteria and had ADHD symptom data available at either the 1-year or 2-year timepoints for the ABCD study sites included in the main analyses (*n* = 9,161) and those included in the lockbox data (*n* = 1,987). Means and standard deviations (SDs) are reported for continuous variables while numbers (*n*) and percentages (%) are reported for categorical variables. *p*(mis) **=** proportion of data missing; HS = high school; GED = General Educational Development Test, equivalent to a high school diploma; * = variable is a *z*-scored aggregate measure that is re-calculated within each training fold

|  | **Main Data** | | | **Lockbox Data** | | |
| --- | --- | --- | --- | --- | --- | --- |
| **Feature** | **Mean/*n*** | **SD/%** | ***p*(mis)** | **Mean/*n*** | **SD/%** | ***p*(mis)** |
| Age (months) | 118.88 | 7.62 | 0.00 | 119.39 | 7.00 | 0.00 |
| Sex - *Male* | 4832 | 53% | 0.00 | 1013 | 51% | 0.00 |
| Sex - *Female* | 4329 | 47% |  | 974 | 49% |  |
| Race - *White* | 5961 | 66% | 0.01 | 1199 | 62% | 0.02 |
| Race - *Black* | 1406 | 16% |  | 275 | 14% |  |
| Race - *Asian* | 213 | 2% |  | 41 | 2% |  |
| Race - *Other/Mixed* | 1490 | 16% |  | 409 | 21% |  |
| Ethnicity - *Non-Hispanic* | 7406 | 82% | 0.01 | 1401 | 71% | 0.01 |
| Ethnicity - *Hispanic* | 1639 | 18% |  | 565 | 29% |  |
| Parents *Married* | 6284 | 69% | <0.01 | 1323 | 67% | 0.01 |
| Parents *Unmarried* | 2818 | 31% |  | 645 | 33% |  |
| Income *<$50k* | 1025 | 23% | 0.08 | 593 | 33% | 0.08 |
| Income >*$50k & <$100k* | 1303 | 29% |  | 533 | 29% |  |
| Income *>$100k* | 2217 | 49% |  | 697 | 38% |  |
| Educ. - *<HS* | 372 | 4% | <0.01 | 152 | 8% | <0.01 |
| Educ. - *HS/GED* | 837 | 9% |  | 181 | 9% |  |
| Educ. - *Some College* | 2251 | 25% |  | 586 | 30% |  |
| Educ. - *Bachelor* | 2398 | 26% |  | 476 | 24% |  |
| Educ. - *Post-Grad.* | 3296 | 36% |  | 586 | 30% |  |
| Area Deprivation Index | * | * | 0.05 | * | * | 0.04 |
| Area total crimes | 9727.60 | 18430.90 | 0.05 | 11420.86 | 7479.42 | 0.04 |
| Area HS graduation rate | 76.20 | 16.48 | 0.09 | 80.81 | 11.76 | 0.08 |
| Area 3^rd^ grade math proficiency | 231.01 | 72.39 | 0.10 | 236.27 | 75.27 | 0.08 |
| Area 3^rd^ grade reading prof. | 212.17 | 68.78 | 0.10 | 223.37 | 70.76 | 0.08 |
| Area school poverty | 47.67 | 27.15 | 0.09 | 55.24 | 27.18 | 0.08 |
| Area high-quality preschools | -0.10 | 5.06 | 0.09 | 0.71 | 3.68 | 0.08 |
| Area estimated lead risk (1-10) | 4.99 | 3.11 | 0.05 | 5.38 | 3.06 | 0.04 |
| Waist circumference (inches) | 26.41 | 4.20 | <0.01 | 26.45 | 4.53 | <0.01 |
| Body mass index (BMI) | 18.74 | 4.22 | <0.01 | 19.07 | 4.19 | 0.01 |

**Supplemental Table 4**. Descriptive statistics and missing data proportions for child self-report variables used as features in the predictive models. Values are from all individuals who met basic inclusion criteria and had ADHD symptom data available at either the 1-year or 2-year timepoints for the ABCD study sites included in the main analyses (*n* = 9,161) and those included in the lockbox data (*n* = 1,987). Means and standard deviations (SDs) are reported for all continuous variables. *p*(mis) **=** proportion of data missing; UPPS = Impulsive Behavior Scale; BIS = Behavioral Inhibition System; BAS = Behavioral Approach System

|  | **Main Data** | | | **Lockbox Data** | | |
| --- | --- | --- | --- | --- | --- | --- |
| **Feature** | **Mean** | **SD** | ***p*(mis)** | **Mean** | **SD** | ***p*(mis)** |
| Neighborhood crime/safety | 4.06 | 1.08 | <0.01 | 3.95 | 1.11 | <0.01 |
| School environment | 3.32 | 0.47 | <0.01 | 3.33 | 0.47 | <0.01 |
| School involvement | 3.27 | 0.59 | <0.01 | 3.26 | 0.60 | <0.01 |
| School disengagement | 1.87 | 0.73 | <0.01 | 1.88 | 0.74 | <0.01 |
| Family conflict | 2.04 | 1.96 | <0.01 | 1.99 | 1.89 | <0.01 |
| Parental monitoring | 4.39 | 0.51 | <0.01 | 4.36 | 0.55 | <0.01 |
| Weekday screen time | 3.47 | 3.09 | <0.01 | 3.28 | 2.99 | <0.01 |
| Weekend screen time | 4.63 | 3.62 | <0.01 | 4.37 | 3.43 | <0.01 |
| BIS summary | 9.50 | 3.74 | <0.01 | 9.53 | 3.74 | <0.01 |
| BAS reward responsiveness | 10.99 | 2.92 | <0.01 | 11.01 | 2.92 | <0.01 |
| BAS drive | 4.13 | 3.06 | <0.01 | 4.09 | 3.05 | <0.01 |
| BAS fun seeking | 5.67 | 2.64 | <0.01 | 5.80 | 2.65 | <0.01 |
| UPPS negative urgency | 8.45 | 2.63 | <0.01 | 8.62 | 2.66 | <0.01 |
| UPPS lack of planning | 7.73 | 2.38 | <0.01 | 7.78 | 2.31 | <0.01 |
| UPPS sensation seeking | 9.78 | 2.67 | <0.01 | 9.69 | 2.68 | <0.01 |
| UPPS positive urgency | 7.93 | 2.95 | <0.01 | 8.20 | 2.98 | <0.01 |
| UPPS lack of perseverance | 7.04 | 2.24 | <0.01 | 7.05 | 2.28 | <0.01 |

**Supplemental Table 5**. Descriptive statistics and missing data proportions for neurocognitive variables used as features in the predictive models. Values are from all individuals who met basic inclusion criteria and had ADHD symptom data available at either the 1-year or 2-year timepoints for the ABCD study sites included in the main analyses (*n* = 9,161) and those included in the lockbox data (*n* = 1,987). Means and standard deviations (SDs) are reported for continuous variables while numbers (*n*) and percentages (%) are reported for categorical variables. *p*(mis) **=** proportion of data missing; NIHTB = NIH Toolbox; RAVLT = Rey Auditory Verbal Learning Test; WISC = Wechsler Intelligence Scale for Children; SST = stop signal task (“go” trials only)

|  | **Main Data** | | | **Lockbox Data** | | |
| --- | --- | --- | --- | --- | --- | --- |
| **Feature** | **Mean/*n*** | **SD/%** | ***p*(mis)** | **Mean/*n*** | **SD/%** | ***p*(mis)** |
| NIHTB picture vocabulary | 107.39 | 17.03 | 0.01 | 105.62 | 16.67 | 0.01 |
| NIHTB flanker | 95.51 | 13.59 | 0.01 | 95.39 | 13.66 | 0.01 |
| NIHTB list sorting | 100.85 | 14.69 | 0.02 | 100.27 | 15.08 | 0.02 |
| NIHTB card sort | 96.98 | 15.24 | 0.01 | 96.14 | 14.84 | 0.01 |
| NIHTB processing speed | 93.89 | 22.16 | 0.01 | 94.10 | 21.37 | 0.01 |
| NIHTB pic. sequence memory | 101.30 | 16.17 | 0.01 | 100.54 | 15.92 | 0.01 |
| NIHTB oral reading | 103.29 | 19.23 | 0.01 | 100.00 | 18.24 | 0.01 |
| WISC matrix reasoning | 9.95 | 2.99 | 0.02 | 9.66 | 2.91 | 0.02 |
| RAVLT total correct recalls | 68.10 | 15.74 | 0.04 | 67.50 | 15.85 | 0.01 |
| Little man task accuracy | 0.59 | 0.17 | 0.03 | 0.60 | 0.17 | 0.03 |
| SST drift rate | 2.24 | 0.60 | 0.21 | 2.18 | 0.59 | 0.22 |
| SST boundary separation | 1.22 | 0.19 | 0.21 | 1.20 | 0.18 | 0.22 |
| SST nondecision time | 0.31 | 0.07 | 0.21 | 0.30 | 0.07 | 0.22 |
| N-back average drift rate | 1.15 | 0.34 | 0.27 | 1.12 | 0.34 | 0.25 |
| N-back drift load effect | 0.46 | 0.40 | 0.27 | 0.47 | 0.43 | 0.25 |
| N-back average boundary sep. | 1.63 | 0.08 | 0.27 | 1.62 | 0.07 | 0.25 |
| N-back boundary load effect | -0.05 | 0.10 | 0.27 | -0.05 | 0.10 | 0.25 |
| N-back average nondecision | 0.57 | 0.10 | 0.27 | 0.55 | 0.09 | 0.25 |
| N-back nondecision load effect | -0.08 | 0.09 | 0.27 | -0.08 | 0.09 | 0.25 |
| Cash Choice – *Delayed* | 5377 | 60% | 0.02 | 1199 | 61% | 0.01 |
| Cash Choice - *Immediate* | 3609 | 40% |  | 763 | 39% |  |

**Supplemental Figure 1**. Scatterplot with linear regression line comparing cross-rater ADHD symptoms (Sx.) factor scores in 1,000 participants at the 1-year timepoint estimated with their complete empirical teacher-report data (x-axis) versus estimated with their teacher-report data artificially treated as missing and replaced with multiple imputation (y-axis).

**
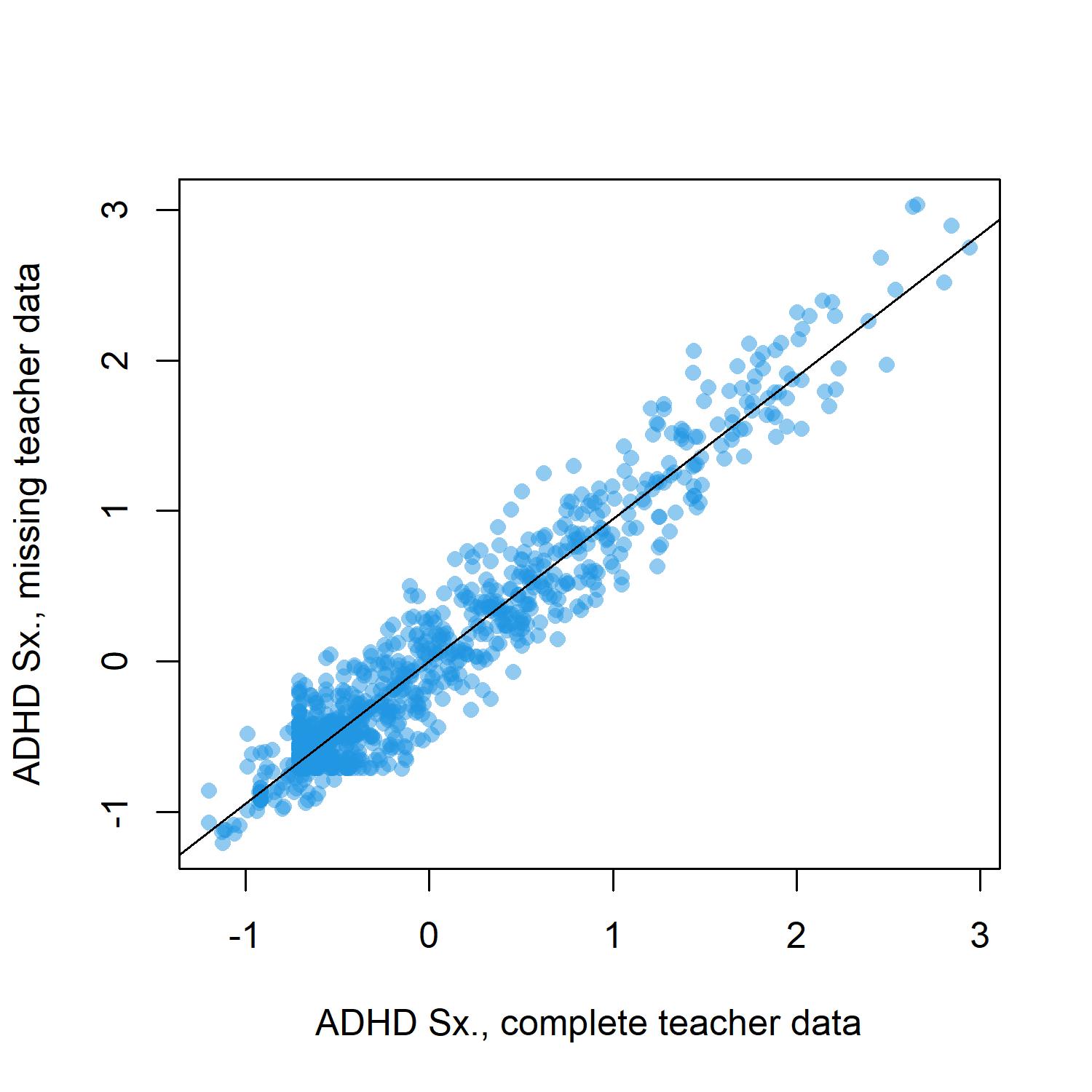
**

**Supplemental Figure 2**. Posterior predictive plots of the DDM’s description of stop-signal task (SST) data from three randomly selected ABCD sites. Plots display, in each stimulus condition, the average cumulative probabilities of a “left” (solid line) or “right” (dotted line) response for empirical data (bold lines) and data predicted by the model (thin lines). Hollow dots represent the empirical 0.1, 0.3, 0.5, 0.7, and 0.9 RT quantiles while solid dots represent the corresponding predicted quantiles. Small gray dots represent the quantiles predicted by 100 separate draws from the posterior distribution and therefore gauge uncertainty in the model predictions.


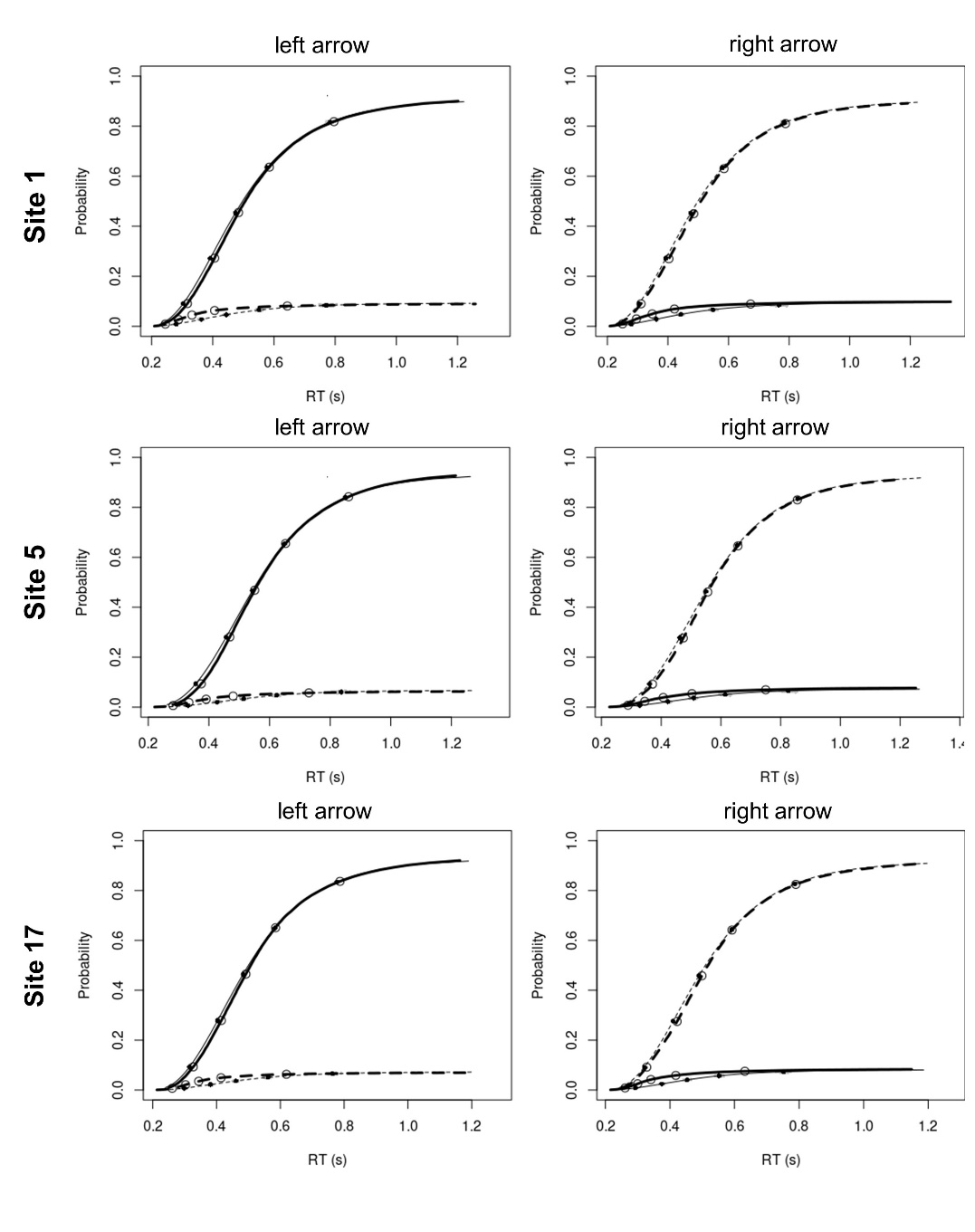


**Supplemental Figure 3**. Posterior predictive plots of the DDM’s description of 0-back data from three randomly selected ABCD sites. Plots display, in each stimulus condition, the average cumulative probabilities of a “non-target” (solid line) or “target” (dotted line) response for empirical data (bold lines) and data predicted by the model (thin lines). Hollow dots represent the empirical 0.1, 0.3, 0.5, 0.7, and 0.9 RT quantiles while solid dots represent the corresponding predicted quantiles. Small gray dots represent the quantiles predicted by 100 separate draws from the posterior distribution and therefore gauge uncertainty in the model predictions.


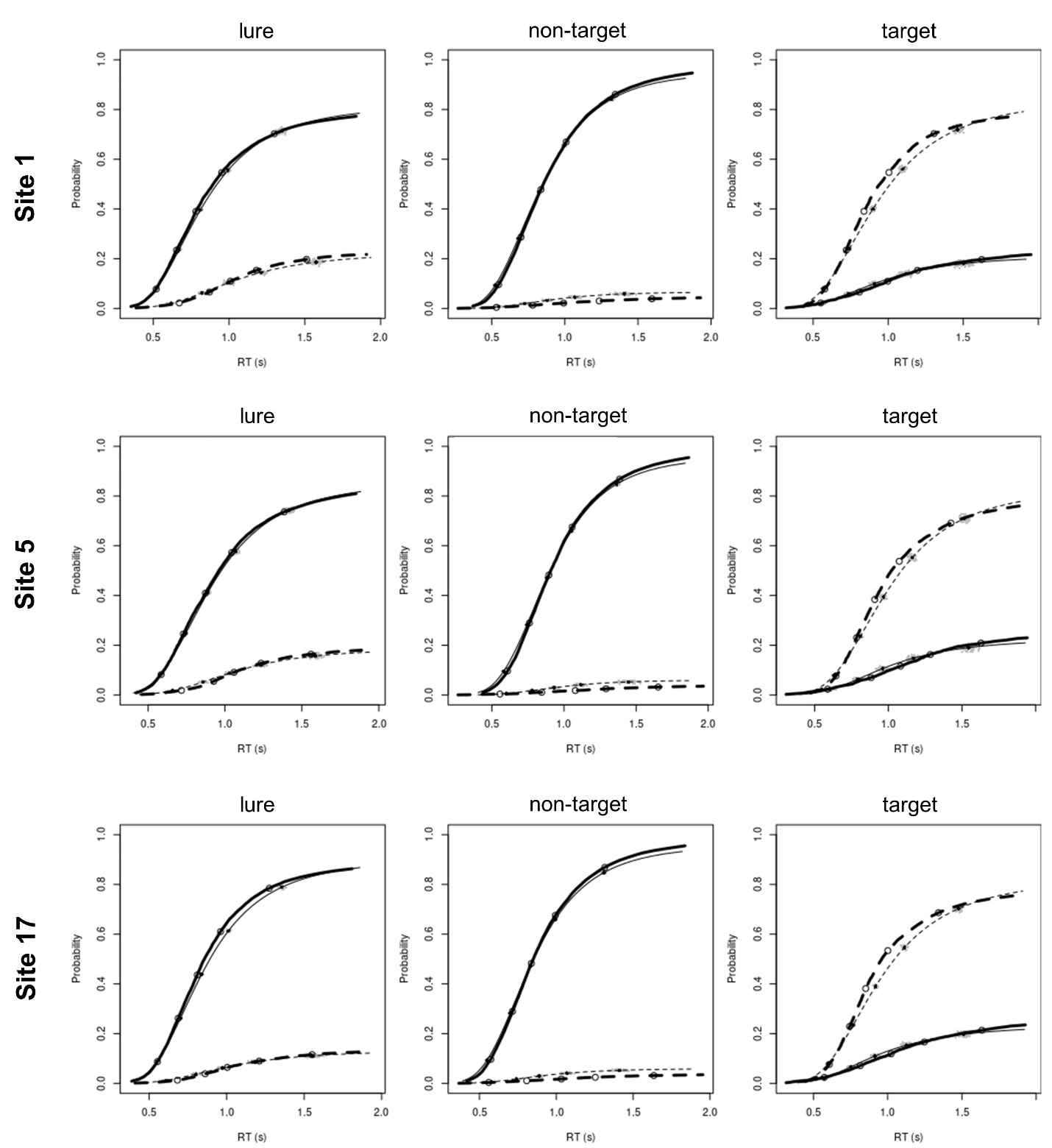


**Supplemental Figure 4**. Posterior predictive plots of the DDM’s description of 2-back data from three randomly selected ABCD sites. Plots display, in each stimulus condition, the average cumulative probabilities of a “non-target” (solid line) or “target” (dotted line) response for empirical data (bold lines) and data predicted by the model (thin lines). Hollow dots represent the empirical 0.1, 0.3, 0.5, 0.7, and 0.9 RT quantiles while solid dots represent the corresponding predicted quantiles. Small gray dots represent the quantiles predicted by 100 separate draws from the posterior distribution and therefore gauge uncertainty in the model predictions.


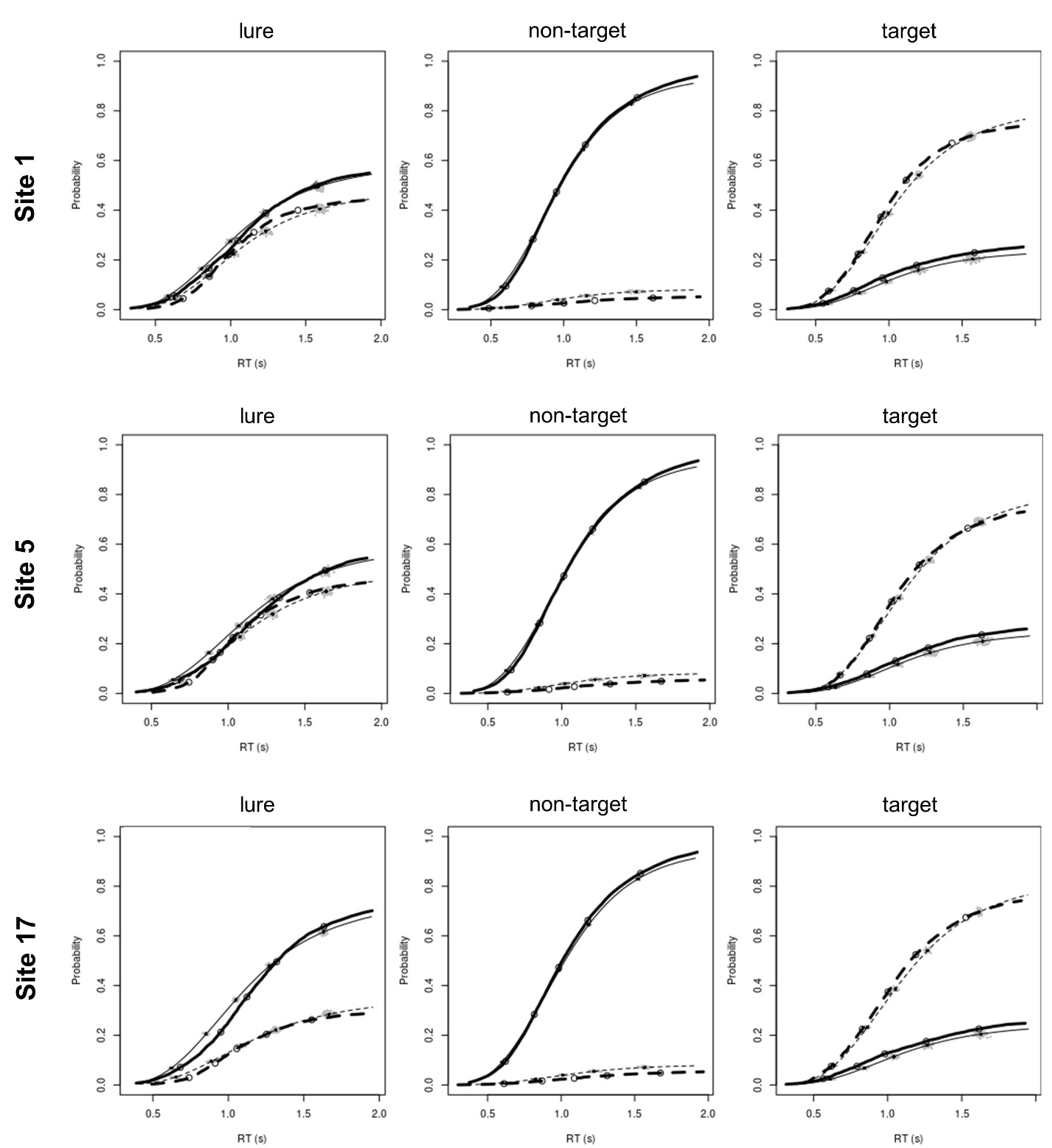


**Supplemental Figure 5**. Feature weights, averaged across training folds and ranked by absolute value, for the comprehensive PCR models and the sparse LASSO models predicting ADHD symptoms at the 1-year timepoint in the sample of individuals who had no missing ADHD symptom report data at that time point. The sparse LASSO model only included 14 of the most relevant features (i.e., those consistently selected in all training folds as well as both halves of the split-half analysis). Weights are color-coded by variable domain: blue = demographic/geocoded/biometric, red = child self-report, orange = neurocognitive testing. Continuous features are shown separately from categorical features because the predictor variables were scaled differently; continuous features were *z*-scored (mean = 0, SD = 1) while categorical features were coded as dummy variables with 0 = reference group versus 1 = feature present. RAVLT = Rey Auditory Verbal Learning Test; NIHTB = NIH Toolbox; WISC = Wechsler Intelligence Scale for Children; SST = Stop Signal Task; UPPS = Impulsive Behavior Scale; BIS = Behavioral Inhibition System; BAS = Behavioral Approach System; HS = High School; BMI = body mass index


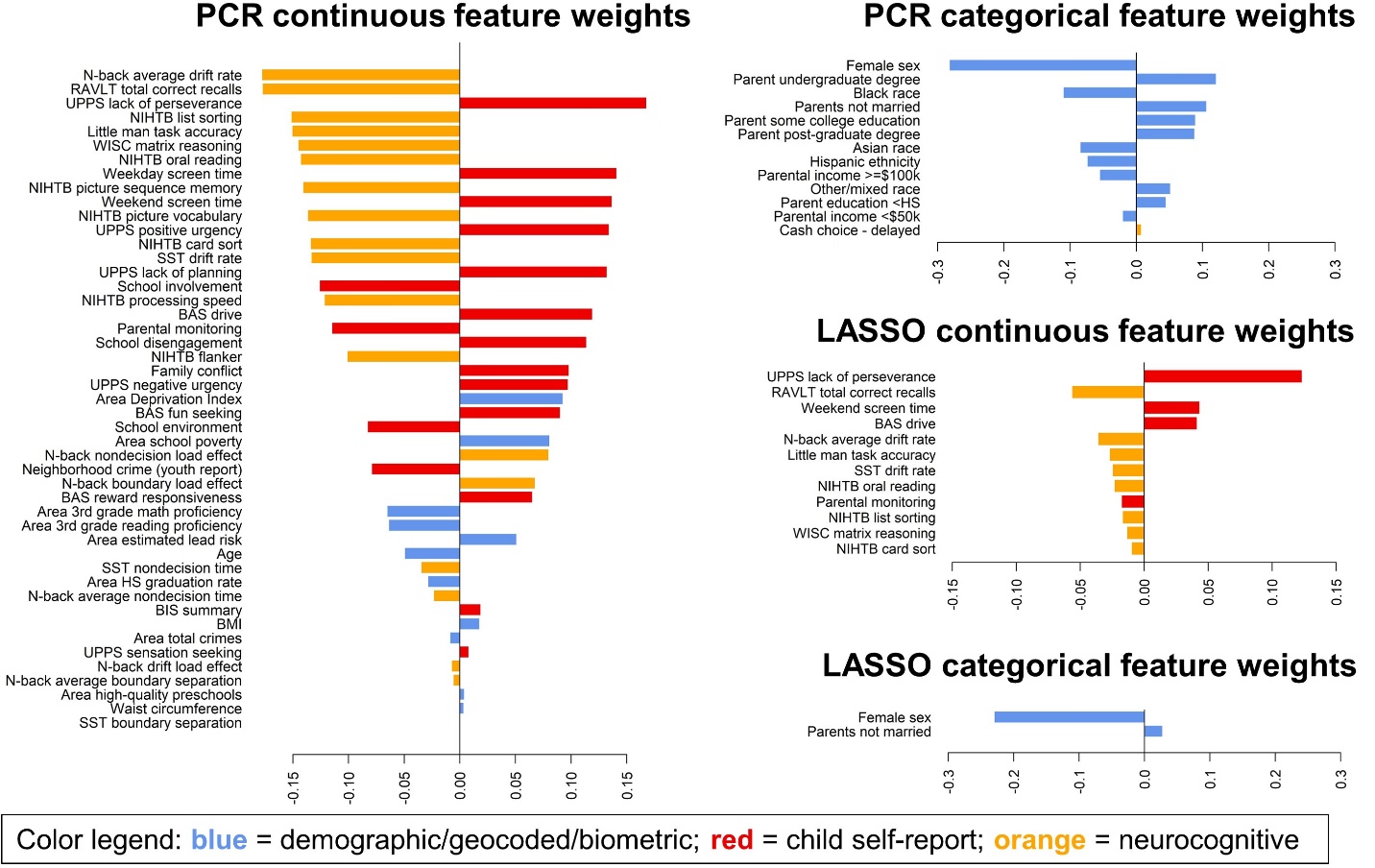


**Supplemental Figure 6**. Feature weights, averaged across training folds and ranked by absolute value, for the comprehensive PCR models and the sparse LASSO models predicting ADHD symptoms at the 2-year timepoint in the full sample with missing symptom data imputed. The sparse LASSO model only included 13 of the most relevant features (i.e., those consistently selected in all training folds as well as both halves of the split-half analysis). Weights are color-coded by variable domain: blue = demographic/geocoded/biometric, red = child self-report, orange = neurocognitive testing. Continuous features are shown separately from categorical features because the predictor variables were scaled differently; continuous features were *z*-scored (mean = 0, SD = 1) while categorical features were coded as dummy variables with 0 = reference group versus 1 = feature present. RAVLT = Rey Auditory Verbal Learning Test; NIHTB = NIH Toolbox; WISC = Wechsler Intelligence Scale for Children; SST = Stop Signal Task; UPPS = Impulsive Behavior Scale; BIS = Behavioral Inhibition System; BAS = Behavioral Approach System; HS = High School; BMI = body mass index


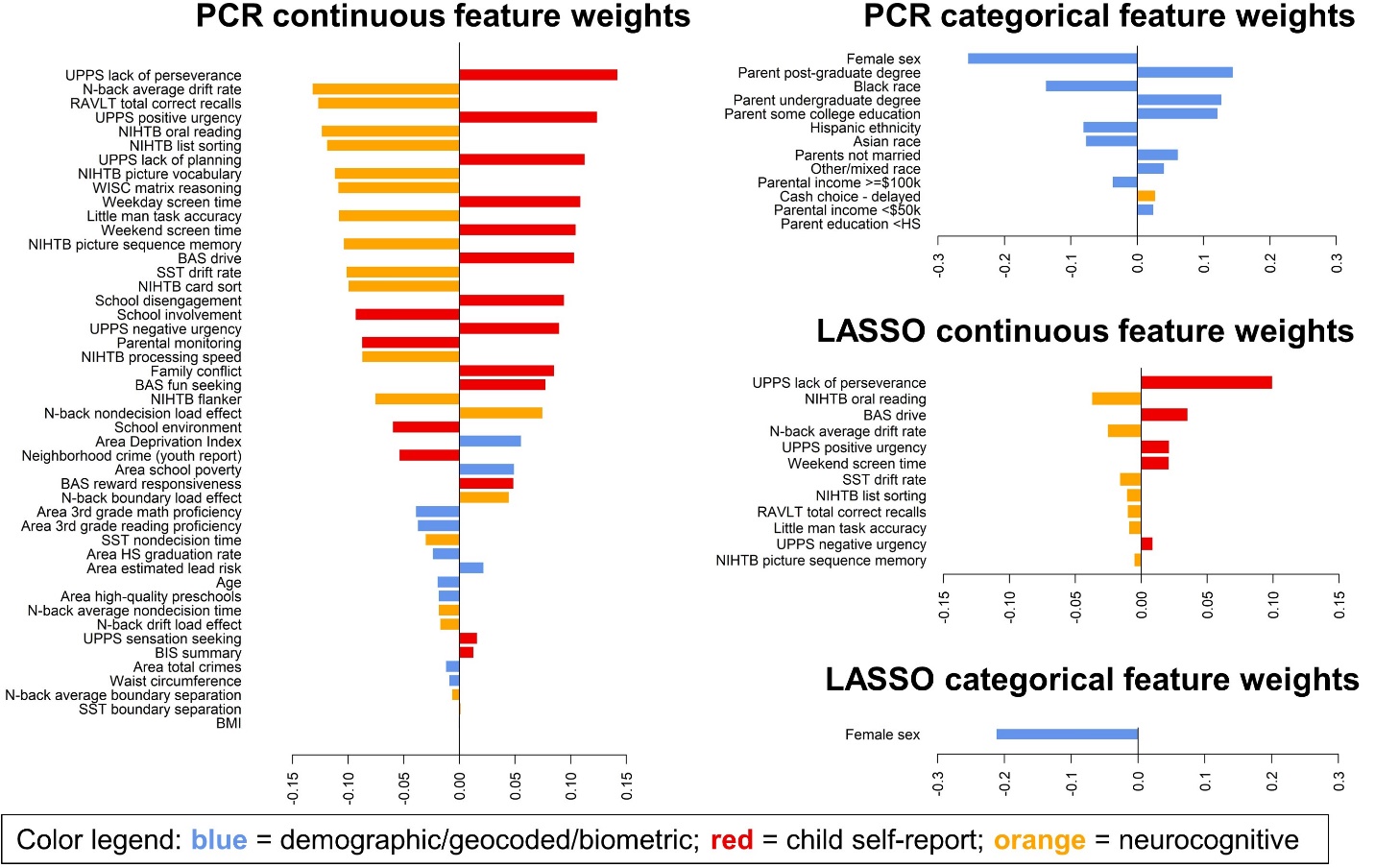


**Supplemental Figure 7**. Feature weights, averaged across training folds and ranked by absolute value, for the comprehensive PCR models and the sparse LASSO models predicting changes in ADHD symptoms from the baseline timepoint to the 2-year timepoint in the full sample with missing symptom data imputed. The sparse LASSO model only included 7 of the most relevant features (i.e., those consistently selected in all training folds as well as both halves of the split-half analysis). Weights are color-coded by variable domain: blue = demographic/geocoded/biometric, red = child self-report, orange = neurocognitive testing. Continuous features are shown separately from categorical features because the predictor variables were scaled differently; continuous features were *z*-scored (mean = 0, SD = 1) while categorical features were coded as dummy variables with 0 = reference group versus 1 = feature present. RAVLT = Rey Auditory Verbal Learning Test; NIHTB = NIH Toolbox; WISC = Wechsler Intelligence Scale for Children; SST = Stop Signal Task; UPPS = Impulsive Behavior Scale; BIS = Behavioral Inhibition System; BAS = Behavioral Approach System; HS = High School; BMI = body mass index


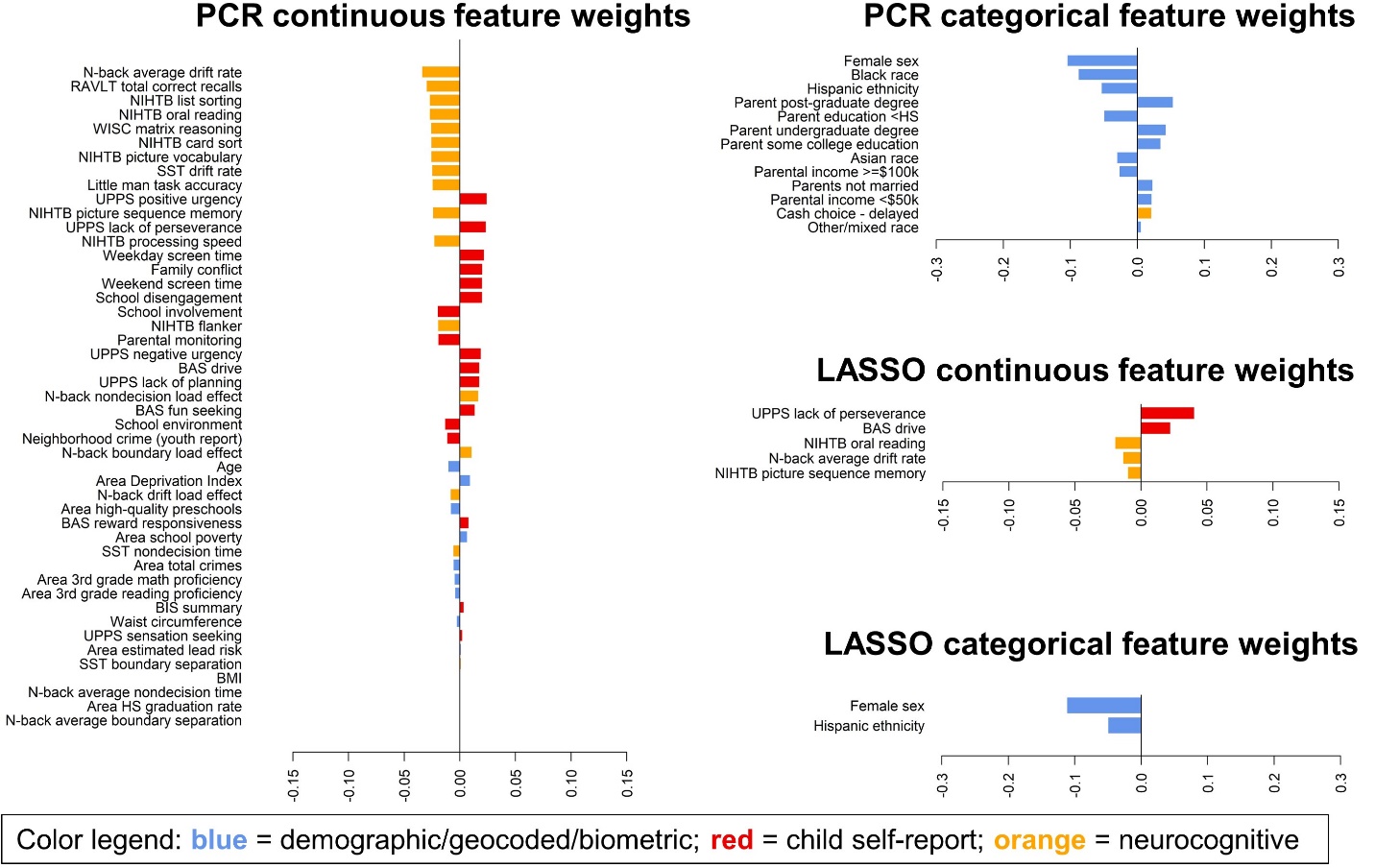


**Supplemental References**

1 Giordano C, Ones DS, Waller NG, Stanek KC. Exploratory bifactor measurement models in vocational behavior research. *Journal of Vocational Behavior* 2020; **120**: 103430.

2 Rosseel Y. Lavaan: An R package for structural equation modeling and more. Version 0.5–12 (BETA). *Journal of statistical software* 2012; **48**: 1–36.

3 Van Buuren S, Groothuis-Oudshoorn K. mice: Multivariate imputation by chained equations in R. *Journal of statistical software* 2011; **45**: 1–67.

4 Narad ME, Garner AA, Peugh JL, Tamm L, Antonini TN, Kingery KM *et al.* Parent–teacher agreement on ADHD symptoms across development. *Psychological assessment* 2015; **27**: 239.

5 Fan CC, Marshall A, Smolker H, Gonzalez MR, Tapert SF, Barch DM *et al.* Adolescent Brain Cognitive Development (ABCD) study Linked External Data (LED): Protocol and practices for geocoding and assignment of environmental data. *Developmental cognitive neuroscience* 2021; **52**: 101030.

6 Taylor RL, Cooper SR, Jackson JJ, Barch DM. Assessment of neighborhood poverty, cognitive function, and prefrontal and hippocampal volumes in children. *JAMA network open* 2020; **3**: e2023774–e2023774.

7 Carver CS, White TL. Behavioral inhibition, behavioral activation, and affective responses to impending reward and punishment: the BIS/BAS scales. *Journal of personality and social psychology* 1994; **67**: 319.

8 Barch DM, Albaugh MD, Avenevoli S, Chang L, Clark DB, Glantz MD *et al.* Demographic, physical and mental health assessments in the adolescent brain and cognitive development study: Rationale and description. *Developmental cognitive neuroscience* 2018; **32**: 55–66.

9 Moos RH. *Family environment scale manual: Development, applications, research*. Consulting Psychologists Press, 1994.

10 Zucker RA, Gonzalez R, Ewing SWF, Paulus MP, Arroyo J, Fuligni A *et al.* Assessment of culture and environment in the Adolescent Brain and Cognitive Development Study: Rationale, description of measures, and early data. *Developmental cognitive neuroscience* 2018; **32**: 107–120.

11 Gonzalez R, Thompson EL, Sanchez M, Morris A, Gonzalez MR, Ewing SWF *et al.* An update on the assessment of culture and environment in the ABCD Study®: Emerging literature and protocol updates over three measurement waves. *Developmental cognitive neuroscience* 2021; **52**: 101021.

12 Chilcoat HD, Anthony JC. Impact of parent monitoring on initiation of drug use through late childhood. *Journal of the American Academy of Child & Adolescent Psychiatry* 1996; **35**: 91–100.

13 Luciana M, Bjork J, Nagel B, Barch D, Gonzalez R, Nixon S *et al.* Adolescent neurocognitive development and impacts of substance use: Overview of the adolescent brain cognitive development (ABCD) baseline neurocognition battery. *Developmental cognitive neuroscience* 2018; **32**: 67–79.

14 Weintraub S, Dikmen SS, Heaton RK, Tulsky DS, Zelazo PD, Bauer PJ *et al.* Cognition assessment using the NIH Toolbox. *Neurology* 2013; **80**: S54–S64.

15 Eriksen BA, Eriksen CW. Effects of noise letters upon the identification of a target letter in a nonsearch task. *Perception & psychophysics* 1974; **16**: 143–149.

16 Schmidt M, others. *Rey auditory verbal learning test: A handbook*. Western Psychological Services Los Angeles, CA, 1996.

17 Wechsler D. Wechsler Intelligence Scale for Children-(WISC-V) Bloomington. *MN: Pearson Education* 2014.

18 Acker WL, Acker C. *Bexley Maudsley Automated Psychological Screening and Bexley Maudsley Category Sorting Test Manual*. NFER-Nelson, for the Institute of Psychiatry, 1982.

19 Ratcliff R. A theory of memory retrieval. *Psychological review* 1978; **85**: 59.

20 Ratcliff R, Smith PL, Brown SD, McKoon G. Diffusion Decision Model: Current Issues and History. *Trends Cogn Sci* 2016; **20**: 260–281.

21 Lerche V, Voss A, Nagler M. How many trials are required for parameter estimation in diffusion modeling? A comparison of different optimization criteria. *Behavior research methods* 2017; **49**: 513–537.

22 Casey B, Cannonier T, Conley MI, Cohen AO, Barch DM, Heitzeg MM *et al.* The adolescent brain cognitive development (ABCD) study: imaging acquisition across 21 sites. *Developmental cognitive neuroscience* 2018; **32**: 43–54.

23 Karalunas SL, Huang-Pollock CL, Nigg JT. Decomposing attention-deficit/hyperactivity disorder (ADHD)-related effects in response speed and variability. *Neuropsychology* 2012; **26**: 684.

24 Fosco WD, White CN, Hawk LW. Acute stimulant treatment and reinforcement increase the speed of information accumulation in children with ADHD. *Journal of abnormal child psychology* 2017; **45**: 911–920.

25 Evans NJ, Steyvers M, Brown SD. Modeling the covariance structure of complex datasets using cognitive models: An application to individual differences and the heritability of cognitive ability. *Cognitive science* 2018; **42**: 1925–1944.

26 Pedersen ML, Alnæs D, van der Meer D, Fernandez-Cabello S, Berthet P, Dahl A *et al.* Computational modeling of the N-Back task in the ABCD study: associations of drift diffusion model parameters to polygenic scores of mental disorders and cardiometabolic diseases. *Biological Psychiatry: Cognitive Neuroscience and Neuroimaging* 2022.

27 Schmiedek F, Li S-C, Lindenberger U. Interference and facilitation in spatial working memory: age-associated differences in lure effects in the n-back paradigm. *Psychology and aging* 2009; **24**: 203.

28 Damaso KA, Castro SC, Todd J, Strayer DL, Provost A, Matzke D *et al.* A cognitive model of response omissions in distraction paradigms. *Memory & Cognition* 2021; : 1–17.

29 Turner BM, Sederberg PB, Brown SD, Steyvers M. A method for efficiently sampling from distributions with correlated dimensions. *Psychological methods* 2013; **18**: 368.

30 Heathcote A, Lin Y-S, Reynolds A, Strickland L, Gretton M, Matzke D. Dynamic models of choice. *Behavior research methods* 2019; **51**: 961–985.

31 Weigard A, Matzke D, Tanis C, Heathcote A. Cognitive process modeling addresses context independence violations in the ABCD Study stop-signal task. *bioRxiv* 2021.

32 Gelman A, Meng X-L, Stern H. Posterior predictive assessment of model fitness via realized discrepancies. *Statistica sinica* 1996; : 733–760.

33 Lê S, Josse J, Husson F, others. FactoMineR: an R package for multivariate analysis. *Journal of statistical software* 2008; **25**: 1–18.

34 Friedman J, Hastie T, Tibshirani R. Regularization paths for generalized linear models via coordinate descent. *Journal of statistical software* 2010; **33**: 1.
